# A cleavage product of Polycystin-1 is a mitochondrial matrix protein that affects mitochondria morphology and function when heterologously expressed

**DOI:** 10.1038/s41598-018-20856-6

**Published:** 2018-02-09

**Authors:** Cheng-Chao Lin, Mahiro Kurashige, Yi Liu, Takeshi Terabayashi, Yu Ishimoto, Tanchun Wang, Vineet Choudhary, Ryan Hobbs, Li-Ka Liu, Ping-Hsien Lee, Patricia Outeda, Fang Zhou, Nicholas P. Restifo, Terry Watnick, Haruna Kawano, Shigeo Horie, William Prinz, Hong Xu, Luis F. Menezes, Gregory G. Germino

**Affiliations:** 1 Kidney Disease Branch; National Institute of Diabetes and Digestive and Kidney Disease, National Institutes of Health (NIH), Bethesda, MD USA; 2 Laboratory of Molecular Genetics; National Heart, Lung and Blood Institute, NIH, Bethesda, MD USA; 3 Division of Nephrology and Endocrinology and the Division of CKD Pathophysiology, University of Tokyo Graduate School of Medicine, Tokyo, Japan; 4 Laboratory of Cell and Molecular Biology; National Institute of Diabetes and Digestive and Kidney Disease, NIH, Bethesda, MD USA; 5 Center for Cell-Based Therapy, National Cancer Institute, NIH, Bethesda, MD USA; 6 Department of Medicine, Division of Nephrology, University of Maryland School of Medicine, Baltimore, MD USA; 7 Department of Urology, Juntendo University Graduate School of Medicine, Tokyo, Japan

## Abstract

Recent studies have reported intrinsic metabolic reprogramming in *Pkd1* knock-out cells, implicating dysregulated cellular metabolism in the pathogenesis of polycystic kidney disease. However, the exact nature of the metabolic changes and their underlying cause remains controversial. We show herein that *Pkd1*^*k*^*o*^*/ko*^ renal epithelial cells have impaired fatty acid utilization, abnormal mitochondrial morphology and function, and that mitochondria in kidneys of ADPKD patients have morphological alterations. We further show that a C-terminal cleavage product of polycystin-1 (CTT) translocates to the mitochondria matrix and that expression of CTT in *Pkd1*^*ko/ko*^ cells rescues some of the mitochondrial phenotypes. Using Drosophila to model *in vivo* effects, we find that transgenic expression of mouse CTT results in decreased viability and exercise endurance but increased CO_2_ production, consistent with altered mitochondrial function. Our results suggest that PC1 may play a direct role in regulating mitochondrial function and cellular metabolism and provide a framework to understand how impaired mitochondrial function could be linked to the regulation of tubular diameter in both physiological and pathological conditions.

## Introduction

Kidney function is dependent on the proper structure of its tubule system. Among the genetic diseases that disrupt nephron architecture, Autosomal Dominant Polycystic Kidney Disease (ADPKD; MIM ID’s 173900, 601313, 613095) is the most common. Caused by mutations in either *PKD1* or *PKD2*, ADPKD is a life-threatening condition estimated to affect almost 1/1000^1^ that often results in renal failure by the sixth decade^2^. *PKD1* encodes polycystin-1 (PC1), a large transmembrane protein^3^ that is autocatalytically cleaved into 3,048-aa N-terminal (NTF; ~325 kDa) and 1,254-aa C-terminal fragments (CTF; ~150 kDa) that remain non-covalently associated^4^. PC1 interacts with polycystin-2 (PC2), the *PKD2* gene product, through a C-terminal coiled-coil domain, and this interaction is thought to be required for proper trafficking and function^5,6^. Additional CTF cleavage products containing the cytoplasmic tail (CTT) have also been described, including a variably sized (~17 kDa, ~34 kDa) fragment reportedly triggered by mechanical stimuli and localized to the nucleus^7,8^; and a ~100 kDa ER product (P100) likely including the final 6 transmembrane (TMs) domains^9^.

PC1-PC2 are often described as a receptor-channel complex, allegedly found in focal adhesions^10^, endoplasmic reticulum (ER)^11^ or primary cilia^12^ and associated with various signaling pathways, including calcium^13^, cAMP^14^, Wnt^15^ and mTOR^16^. In addition to its initially described role as a regulator of apoptosis and proliferation^17^, emerging evidence of intrinsic metabolic reprogramming in *Pkd1* knockout cells suggests that the PC1-PC2 complex regulates cellular metabolism^18–21^. The exact nature of the metabolic alterations remains controversial, however, with some groups reporting enhanced glycolysis reminiscent of the Warburg phenomenon^19,21^ and others observing no evidence for a glycolytic switch^20,22^ and/or proposing fatty acid oxidation impairment^20,23^. The link between PC1, cellular metabolism and regulation of tubular diameter also remains elusive.

We now report that a proteolytic product of PC1 localizes to mitochondria matrix and show that its over-expression in heterologous systems can alter mitochondrial structure and function.

## Results

**Figure 1:**
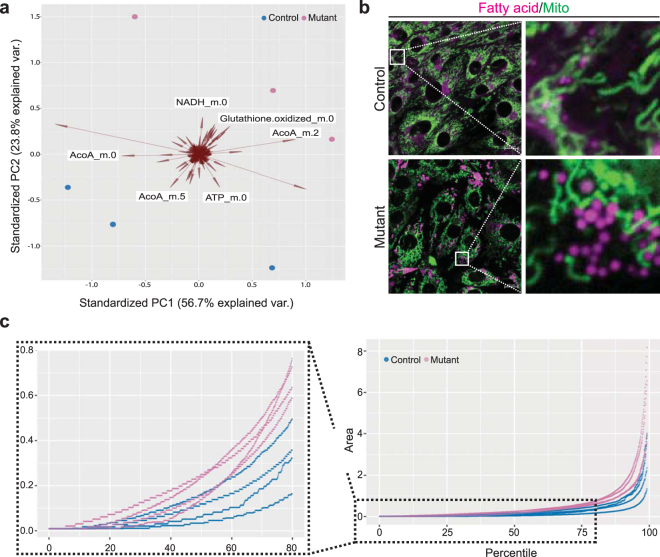
*Pkd1*^*ko/ko*^ cells have metabolic differences. (**a**) Fluxomics. Principal components bi-plot showing clustering of three replicates of a mutant and control immortalized kidney epithelial cell line (94414-LTL) according to flux of ^13^C from labeled glucose through different metabolites. Circles are samples, and their location in the plot is determined by a linear combination of specific factors (metabolites). The direction and weight each metabolite contributes to the location of the sample is represented by the direction and size of the corresponding arrow. Mutant (red circles) and control (blue circles) samples cluster in opposite corners of the figure, and labeled arrows show the metabolites that have the highest influence in separating groups. (**b**) Fatty acid uptake assay showing that mutant cells have increased number and size of lipid droplets (green: mitochondria stained with MitoTracker Green; Magenta: BODIPY 558/568 C_12_). The panels on the right show higher magnification of the areas inside the white squares. (**c**) Quantile plot showing distribution of lipid droplet size quantified in ten random fields in two proximal tubule kidney cell lines (each line is one experiment for one cell line). The insert on the left shows only up to the 80^th^ quantile, to highlight differences within the lower range of area values (n = 4 experiments, p = 0.044; line: percentile values of the lipid droplet area for one experiment, colored by genotype).

*Pkd1* knockout cells have been previously described as metabolically reprogrammed^19–22^. To further investigate a metabolic phenotype in *Pkd1* mutant cells, we analyzed the rate of metabolite turnover – or metabolic flux – by mass spectrometry of cells treated with ^13^C-labeled glucose. In a previously described pair of proximal tubule epithelial kidney cell lines in which the *Pkd1* knockout was derived from its control counterpart (94414-LTL^20^), we confirmed that *Pkd1* inactivation results in a mild, but detectable, shift in metabolite utilization (Fig. 1a, Supplementary Table 1). We have previously reported that *Pkd1* mutant cells have reduced fatty acid metabolism^20^. To further evaluate this abnormality, we investigated fatty acid uptake and utilization by loading cells with labeled lipids. In this assay, *Pkd1* knockout was correlated with increased number and size of lipid droplets, suggesting that lipids were adequately taken up but not utilized as efficiently (Fig. 1b and c, n = 4 experiments, p = 0.044). We next examined whether abnormal fatty acid utilization was accompanied by changes in phospholipid levels. Our results suggest that this is not the case and that the measured biosynthetic pathways are preserved (Supplementary Fig. S1).

**Figure 2:**
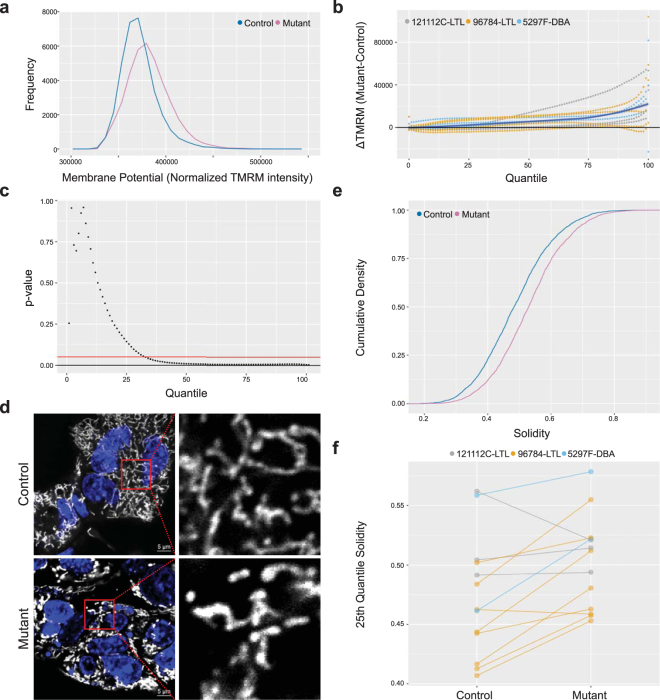
*Pkd1*^*ko/ko*^ cells have differences in mitochondrial function and morphology. (**a**) Representative distribution of mitochondrial membrane potential in 96784-LTL cells measured by flow cytometry and showing increased frequency of mutant cells with higher TMRM fluorescence. (**b**) Curves of the difference between mutant and wild type TMRM fluorescence for each centile of the TMRM distribution, showing that mutant cells tend to have higher TMRM intensity, particularly in the upper centiles. Each dotted line corresponds to one experiment, colored by cell line. The blue solid line is the best fit of the data. (**c**) P-values comparing TMRM intensity at each centile and showing that mutant cells have significantly higher TMRM for cells in the upper two thirds of the TMRM distribution. Red line: p = 0.05, n = 10. (**d**) Representative image showing 96784-LTL control and *Pkd1*^*ko/ko*^ cells stained with mitochondrial marker (gray: MitoTracker Deep Red; blue: Hoechest 33342 nuclear stain). The panels on the right show higher magnification of the areas inside the red squares. Mitochondria form a more elongated and interconnected network in controls. (**e**) Representative cumulative distribution in 96784-LTL control (blue line) and *Pkd1*^*ko/ko*^ cells showing that at most centiles (y-axis) the solidity is higher (i.e. mitochondrial network is more fragmented) in mutants (red line). (**f**) Measure of mitochondrial fragmentation in independent experiments comparing three matched mutant and control cell lines. The y-axis shows the 25^th^ percentile solidity (higher values correspond to more fragmented mitochondrial network). Lines connect matched control (left) and mutant (right) for each independent experiment (n = 13 experiments; average number of analyzed mitochondria/dot: 799; p-value < 0.01).

These results prompted us to investigate PKD mitochondrial function and morphology. Using tetramethylrhodamine, methyl ester (TMRM) fluorescence as readout for mitochondrial membrane potential, we analyzed the effect of *Pkd1* knockout in three independent *Pkd1* conditional cell lines. Compared to their genetically matched controls, mutants showed a small, but significant, increase in TMRM fluorescence (Fig. 2a and b). This effect was most pronounced for cells in the upper half of the distribution (Fig. 2c; p < 0.01 in the 95^th^ quantile, n = 10 experiments). Mitochondria in mutant cells also appeared less elongated (Fig. 2d). The solidity index (Supplementary Fig. S2) has been previously reported as a useful parameter to quantify mitochondrial shape^24^. Using automated measurement of solidity in mitochondrial images, our analyses suggest increased fragmentation of the mitochondrial network (Fig. 2e and f, n = 13 experiments (3 matched cell lines), p < 0.01) in *Pkd1*^*ko/ko*^ cells.

**Figure 3:**
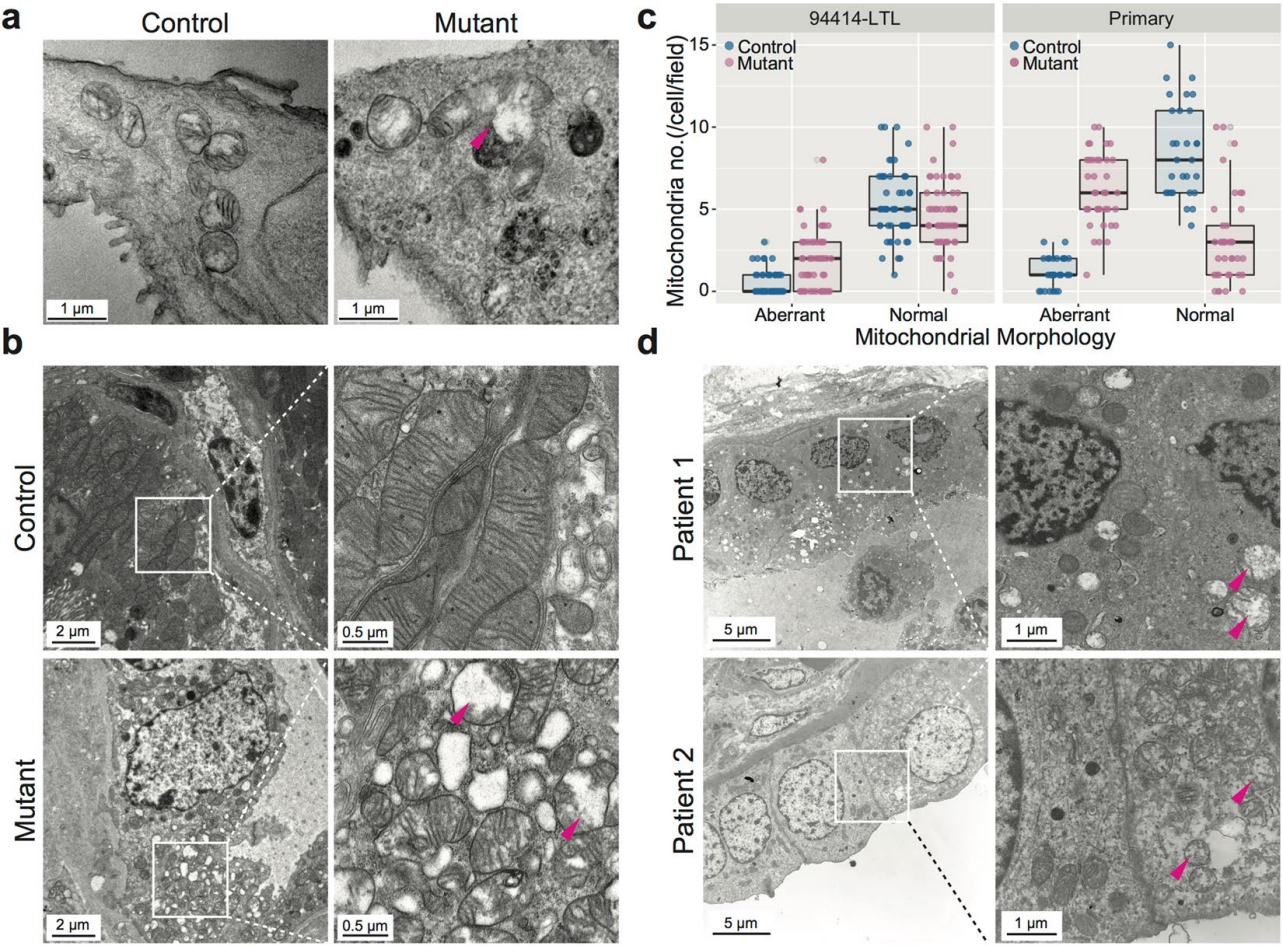
Abnormal mitochondria in mouse and human PKD cells and tissues. (**a**) Electron microscopy showing swollen mitochondria, some with darkly stained membranous whorls, in mutant cells. (**b**) Swollen mitochondria in mutant cells freshly dissociated from cystic and control mouse kidneys. Panels on the right show higher magnification of the areas inside the white squares. (**c**) Quantification of number of “normal” or “aberrant” mitochondria in randomly selected fields of the samples shown in (**a** and **b**); n ≥ 29 fields/condition, p < 0.001. (**d**) Swollen mitochondria in ADPKD human kidney samples. Panels on the right show higher magnification of the areas inside the white squares. Representative aberrant mitochondria are indicated with magenta arrowheads.

Ultrastructural electron microscopy images further corroborated this finding, showing abnormal mitochondria in a proximal tubule-derived cell line (Fig. 3a), in primary cells dissociated from mouse *Pkd1*^*ko/ko*^ kidneys (Fig. 3b and c, n ≥ 29 fields/condition, p < 0.001) and in kidney samples of two ADPKD patients (Fig. 3d).

**Figure 4:**
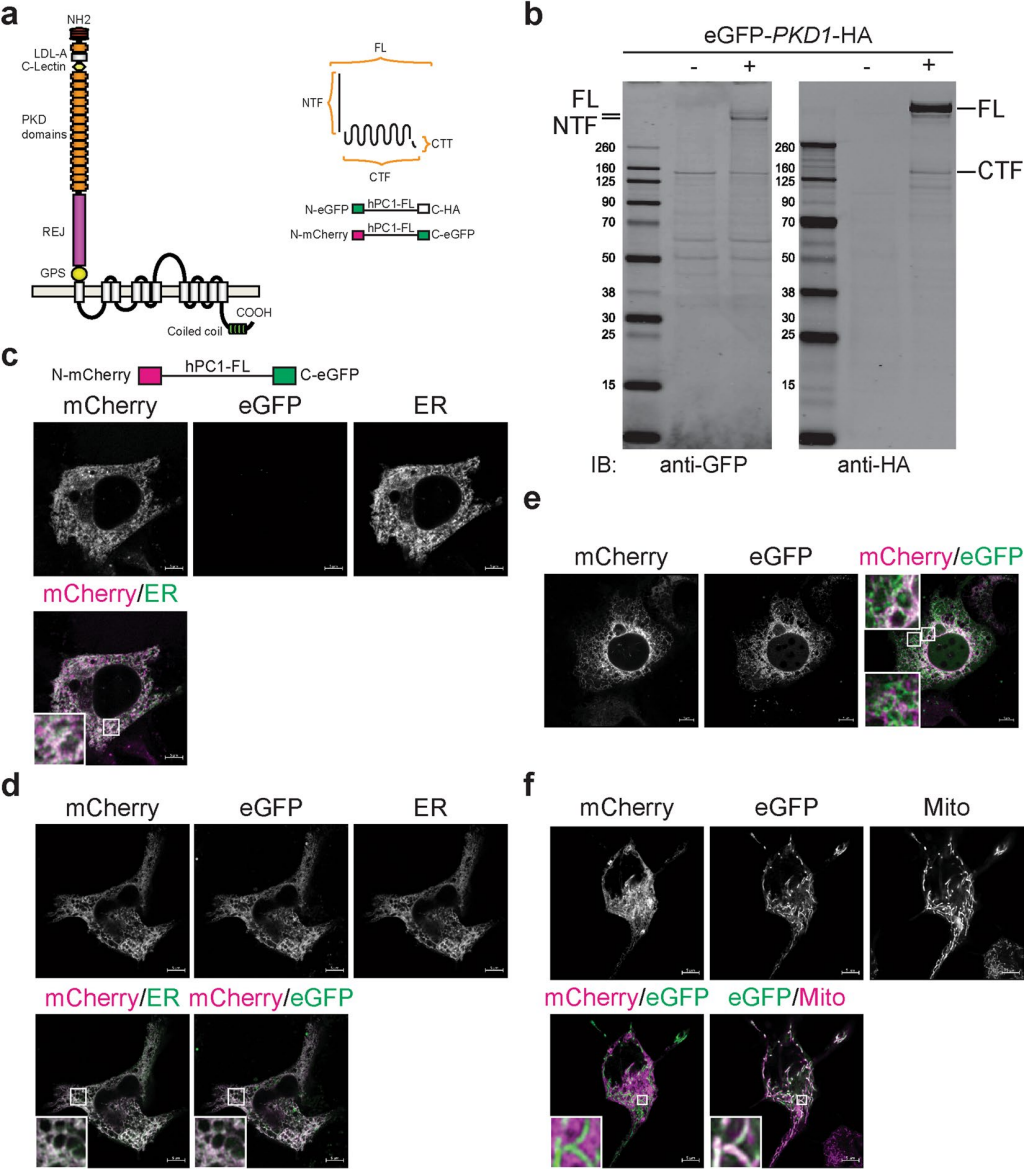
Recombinant PC1 localizes to mitochondria in a subset of cells. (**a**) Schematics showing PC1 domains and predicted topology. Full-length PC1 (FL) is a 4,302-aa protein with 11 predicted transmembrane (TM) domains^3^. N-terminal to the first TM, a G-protein-coupled receptor cleavage site (GPS) undergoes autocatalytic cleavage to produce 3,048-aa N-terminal (NFT; ~325 kDa) and 1,254-aa C-terminal fragments (CTF; ~150 kDa) that remain non-covalently associated^4^. The CTF can be further processed to release a cytoplasmic tail (CTT). Constructs used in the study include full-length human PC1 (hFL) with N-terminal (eGFP or mCherry) and C-terminal (HA or eGFP) tags. (**b**) Lysates from MDCK cells with either stable, inducible expression of vector control (pcDNA5) or eGFP-PKD1-HA constructs probed with GFP or HA antibodies show that the majority of cellular PC1 is free NTF, with only a small amount of uncleaved FL and CTF. (**c**–**f**) NIH3T3 cells transfected with mCherry-PKD1-eGFP show three main patterns: (1) NTF in the ER with undetectable CTF (**c**); (2) FL and/or NTF/CTF either mostly co-localized in the ER (**d**) or with partial co-localization in the ER and with CTF/CTT in distinct subcellular regions (**e**, panel insert); (3) NTF localized to the ER and CTF or CTT in mitochondria (**f**). The endoplasmic reticulum (ER) is identified by transient expression of pEF.myc.ER-E2-Crimson or stained with ER-Tracker™ Blue-White DPX (**c**,**d**) and mitochondria (Mito) are identified by transient expression of mito-BFP or stained with MitoTracker Deep Red.

To gain insights into how polycystin-1 (PC1) could regulate mitochondrial and metabolic functions, we analyzed PC1 subcellular localization using live cell imaging and biochemical methods. PC1 (Fig. 4a) is a large protein with a complicated cleavage pattern, giving rise to various fragments that vary considerably in their relative abundance. In MDCK cells expressing tagged human PC1, the FL is about half as abundant as the NTF, and about 10 times more abundant than CTF, suggesting that there is a large, free pool of NTF not associated with CTF and that CTF/CTT are less stable (Fig. 4b).

To further understand this discrepancy, we did live cell imaging in NIH3T3 cells expressing human PC1 with mCherry added to the N-terminus just after the signal peptide and eGFP added to the C-terminus. Our results show three distinct distribution patterns. In the majority of cells, only the N-term tag is visible and it is present predominantly in the ER (Fig. 4c). This finding is consistent with the data in Fig. 4b that suggest there is a large, free pool of NTF that is far in excess in the amount of detectable CTF. There is a smaller number of cells that express both the N-term and C-term tags. In these cells, the tags mostly co-localize to the ER (Fig. 4d) but sometimes can be seen segregating to distinct subcellular regions (Fig. 4e). The co-localization of signal either is the result of co-localization of cleaved NTF tethered to CTF and/or from FL, uncleaved PC1. Finally, there is a rare set of cells with the NTF localized to the ER and a distinct mitochondrial pattern for the C-term tag (Fig. 4f). While the co-localization of the N-term and C-term tags is likely due to the reasons just described, the mitochondrial pattern suggests that there is a free pool of CTF and/or CTT that traffics to the mitochondria. Efforts to verify the patterns for endogenous PC1 using antisera that recognize the C-terminus were unsuccessful, presumably due to the very low abundance of the protein and the lack of suitable antisera.

**Figure 5:**
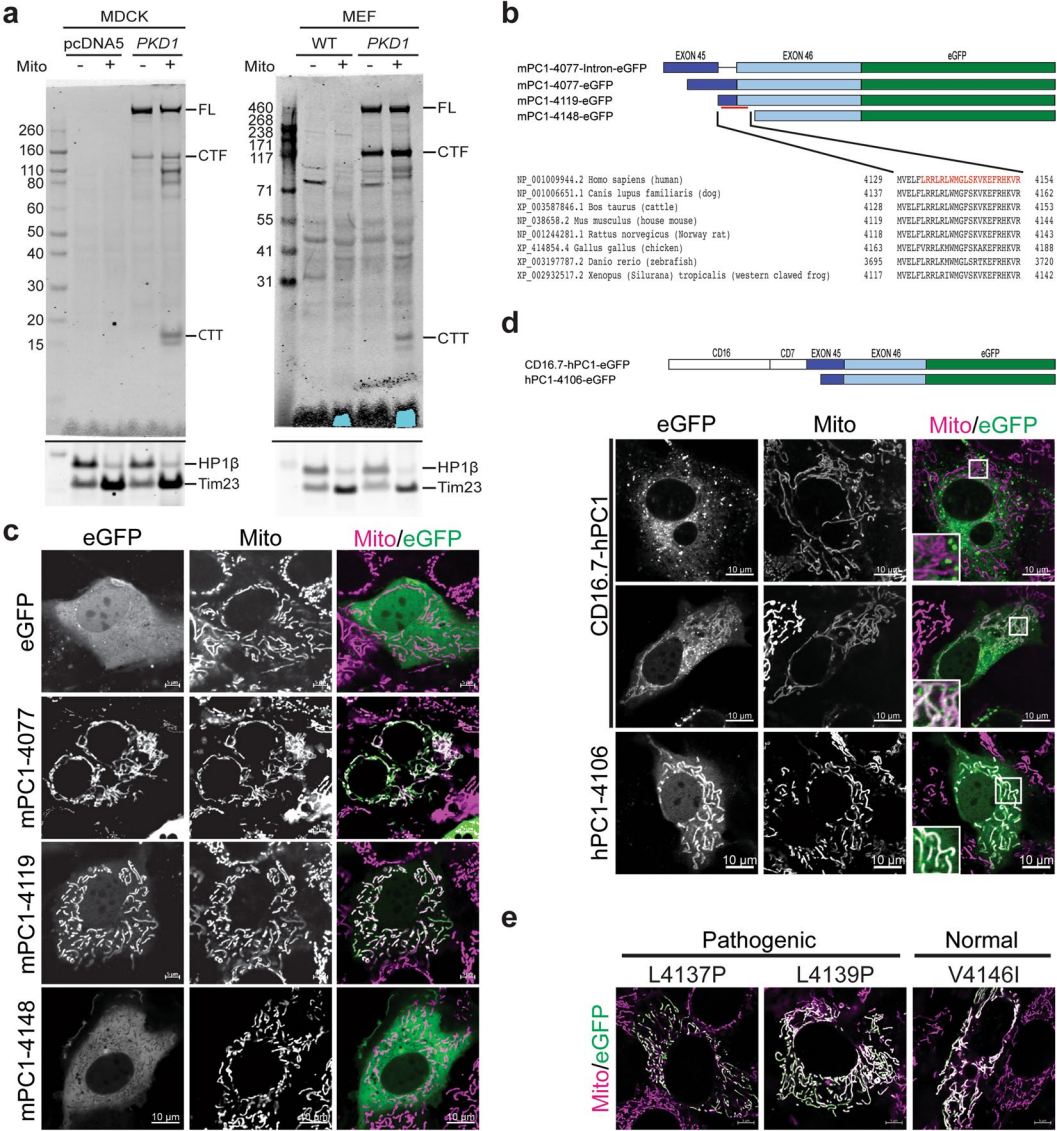
PC1 CTT is enriched in mitochondria. (**a**) Immunoblot showing total cell lysates (“mito−”) and mitochondrial enriched fractions (“mito+”) of MDCK cells with stable, inducible expression of *PKD1* (eGFP-PKD1-HA) or pcDNA5 and of MEFs obtained from wild type or transgenic *Pkd1*^*F/H*^ BAC mice expressing PKD1-HA. While Full-length (FL) and CTF (CTF) are detected in both total lysates and in the mitochondrial fractions, only the PC1 C-terminal fragment (CTT, molecular weight between 15~20 KDa) is consistently enriched in the mitochondrial fraction. The ratio of the lower two bands varied from experiment to experiment (see also Fig. 6a and e, and Supplementary Fig. S3). The cropped blots at the bottom of each panel show subsequent staining of the same membrane with nuclear (HP1β) and mitochondrial (Tim23) markers. (**b**) Constructs of mouse PC1 CTT used in panel “c” to map the putative MTS, a sequence which it is highly conserved. A previously reported nuclear localization sequence (NLS)^7^ is shown in red. (**c**) Fluorescence imaging in live cells expressing eGFP or mPC1-CTT truncation constructs described in panel “b” (Mito: MitoTracker Deep Red). (**d**) NIH3T3 cells transiently transfected with various constructs. Previously published constructs^8^ that express hPC1-CTT either fused to a membrane anchor (CD16.7-h-PC1) or as a smaller truncated form, both including the MTS and NLS sequences. The top panel shows the predominant membrane-anchored pattern of CD16.7-hPC1; the middle panel shows that in some cells this construct is presumably cleaved, releasing a fragment that localizes to mitochondria; in the lower panel the truncated hPC1-CTT is seen predominantly in mitochondria, with diffuse expression elsewhere in the cell but without nuclear enrichment. (**e**) Live cell imaging of human MTS sequence variants fused to eGFP (in green) co-localized with mitotracker (magenta). L4137P and L4139P are predicted to be pathogenic while V4146I is predicted to be a normal variant.

We corroborated the unexpected localization of a PC1-derived C-terminal fragment to mitochondria by immunoblot analysis. Multiple CTF-derived products were enriched in mitochondrial fractions of MDCK cells expressing recombinant PC1 (Fig. 5a). To minimize the likelihood that the mitochondrial pattern was an artifact of over-expression of recombinant constructs, we confirmed the findings in mitochondrial fractions of murine embryonic fibroblasts (MEFs) derived from a mouse line transgenic for a mouse *Pkd1* BAC (bacterial artificial chromosome) that had been tagged with a C-terminal HA tag^25^ (Fig. 5a). While the patterns differed slightly between the cell lines and between experiments, one consistent fragment is of the expected CTT size (Supplementary Fig. S3). These data suggest that PC1 also likely gives rise to a CTT targeted to mitochondria *in vivo* (PC1-CTT).

Using Cas9/CRISPR technology, we sought to generate stable cell clones expressing endogenous PC1 with a C-terminal copGFP tag. After selecting clones for GFP fluorescence assuming this property was an indication of successful knock-in, we instead identified clones with random integration of the knock-in construct that exhibited strong copGFP signal in mitochondria (Supplementary Fig. S4). To exclude the possibility that the pattern was due to properties of the insertion site or genomic elements within the construct, we generated a set of truncation constructs that spanned the length of the targeting construct and used them to map a putative mitochondrial targeting sequence (MTS) that partially overlaps with a previously described nuclear localization sequence (NLS^7^). We found that the MTS was both necessary and sufficient to translocate either PC1-CTT or eGFP to mitochondria, and the results were confirmed in multiple cell lines (NIH3T3, MDCK, mCCD^26^ and mIMCD3; Fig. 5c,e). Because these data differed from what had been previously described for the C-terminus^7,8^, we also tested the previously published construct of human *PKD1* (Fig. 5d). We were able to confirm the mitochondrial pattern and failed to observe nuclear enrichment of the CTT signal. We also failed to see a nuclear pattern for the CTT after treatment with the proteosome inhibitor MG132 (Supplementary Fig. S5). Finally, we sought to exclude the possibility that the unexpected pattern was somehow related to the eGFP tag using constructs that had an HA-epitope substituted for eGFP. We were, however, unable to detect the HA-tagged CTT construct using our standard conditions. We therefore generated a CTT construct harboring both an eGFP and the HA epitope and used this as a positive control to identify permeabilization conditions suitable for detection of both the HA and eGFP (Supplementary Fig. S5). This modified method was then successfully used to show that recombinant CTT-HA lacking eGFP (mPC1-4119-3HA) also localized to mitochondria (Supplementary Fig. S5). Using the same methods, we were unable to detect the HA epitope unambiguously in the *Pkd1*-BAC MEFs, presumably due to its relatively low abundance in these cells.

Several pathogenic (L4137P, L4139P^27^) and normal (V4146I^28^) variants have been reported that modify the MTS sequence. We therefore tested whether any of these disrupted CTT trafficking. After transient expression, all the constructs still exhibited eGFP signal in mitochondria, suggesting that any pathogenic effects associated with these mutations could not be directly attributed to CTT’s inability to translocate to mitochondria (Fig. 5e).

**Figure 6:**
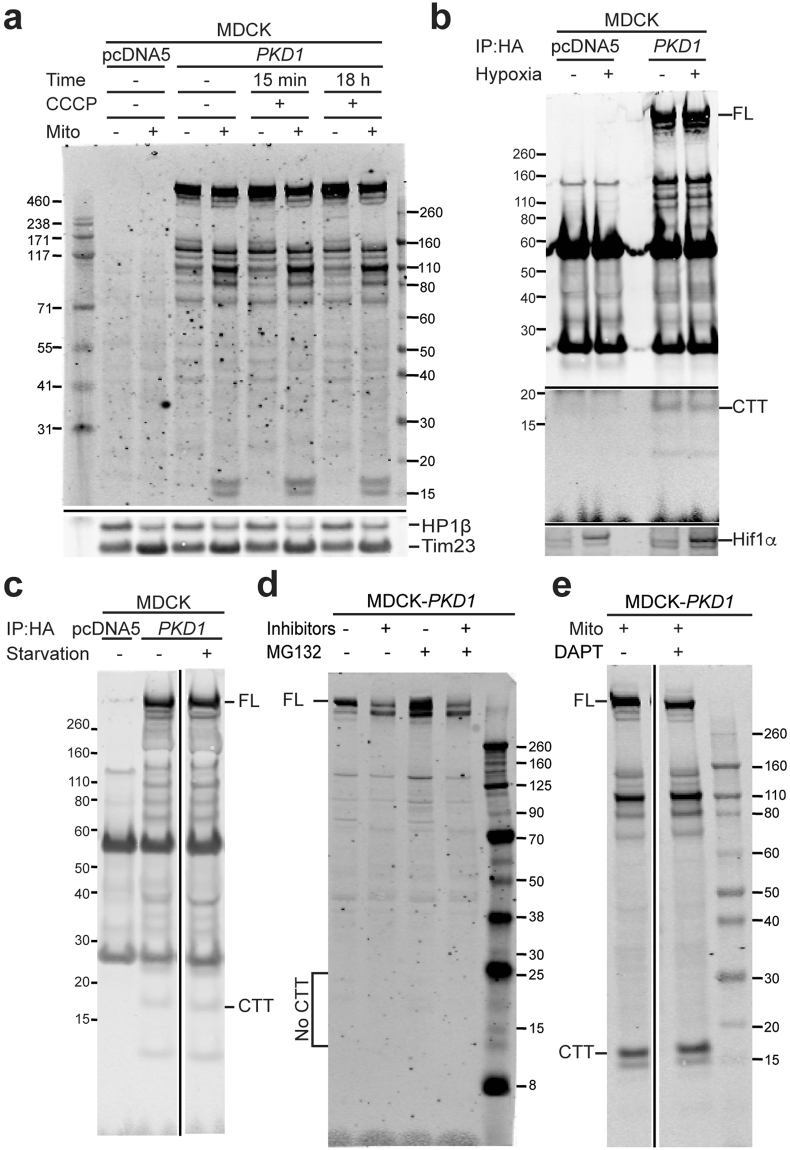
PC1-CTT levels are not altered by cellular stress or inhibition of degradation pathways. (**a**–**c**) Immunoblot of MDCK cells with stable, inducible expression of *PKD1* (eGFP-PKD1-HA) or pcDNA5. PC1-FL and PC1-CTT amounts are unchanged after (**a**) treatment with 1 μM CCCP for 15 min or 18 hours; (**b**) 2 h hypoxia (0.01% O_2_, 5% CO_2_); or (**c**) 48 h serum starvation. The bottom panel in “b” confirms Hif1α induction in the total lysate of the corresponding samples. In panel “b”, the same blot is shown at low signal intensity in the high molecular weight range, and at high signal intensity in the low molecular weight range, to optimize visualization of the relevant bands (original images are presented in Supplementary Fig. S6A). The samples in panels “c” and “e” were run on the same gel for each experiment, with the middle, irrelevant lanes removed for clarity (see original images, Supplementary Fig. S6B and C). (**d**,**e**) 24 h treatment with protease inhibitors (**d**) or γ-secretase inhibitor (**e**) had no effect on PC1-CTT detection.

While CTT is only rarely seen in mitochondria in cells expressing PC1-FL with either a C-terminal HA or eGFP tag, CTT with HA or eGFP tag is overwhelmingly mitochondrial in cells expressing the shorter constructs. We hypothesized that CTT release from CTF was regulated and therefore tested multiple stimuli including carbonyl cyanide m-chlorophenyl hydrazone (CCCP), hypoxia and serum starvation (Fig. 6a–c). We have so far failed to identify chemicals that prevent CTF degradation and/or consistently enhance CTT detection (Fig. 6d). In contrast to a previous report^29^, release of the CTT fragment was not consistently prevented by a γ-secretase inhibitor (Fig. 6e).

**Figure 7:**
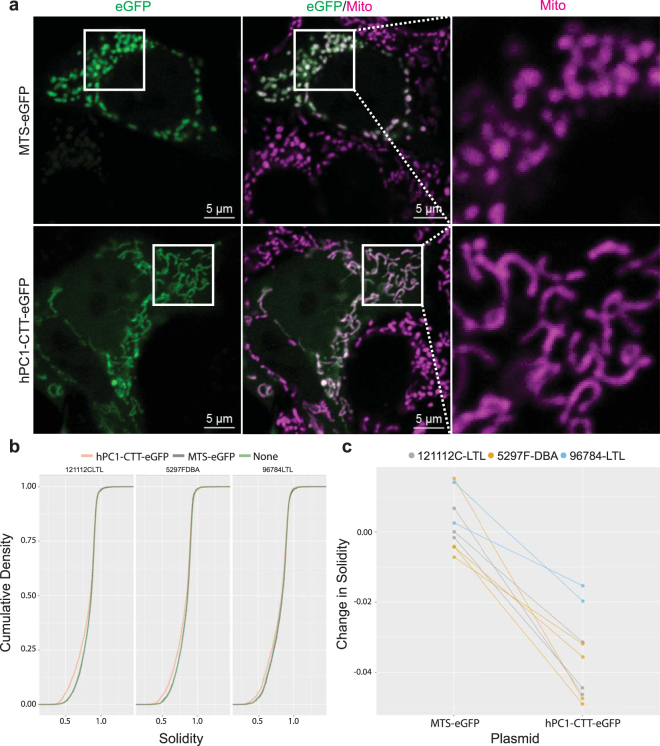
PC1-CTT alters mitochondrial network structure. (**a**) Mutant epithelial cells transfected with hPC1-CTT-eGFP or MTS-eGFP. Transfected cells express eGFP and can be seen in the left panels. The middle panels show mitochondria stained with MitoTracker Deep Red in magenta. The area inside the solid squares is in shown on the right. Note that mitochondria appear more elongated in cells expressing PC1-CTT. (**b**) Cumulative density of solidity index in at least three independent experiments in three different mutant cell lines. Note that cells expressing PC1-CTT have solidity distribution shifted towards lower values. (**c**) For each experiment, the solidity was measured between un-transfected and transfected cells. The change in solidity at the 25^th^ quantile was close to zero in cells expressing MTS-eGFP, but significantly lower in cells expressing PC1-CTT (p < 0.001; each line corresponds to one experiment, colored by cell line).

To test the relationship between CTT and mitochondrial phenotypes, we queried whether its re-expression in *Pkd1* mutant cells could rescue any of their mutant properties. We transiently expressed either PC1-CTT fused to eGFP, or a control plasmid with eGFP targeted to mitochondria (MTS-eGFP) in three independent *Pkd1* mutant renal epithelial cell lines. Mitochondria in cells expressing PC1-CTT appeared more elongated (Fig. 7a), and quantification of solidity indices confirmed that presence of PC1-CTT correlated with decreased solidity (Fig. 7b and c; n = 2 to 5 experiments/cell line; p < 0.001).

**Figure 8:**
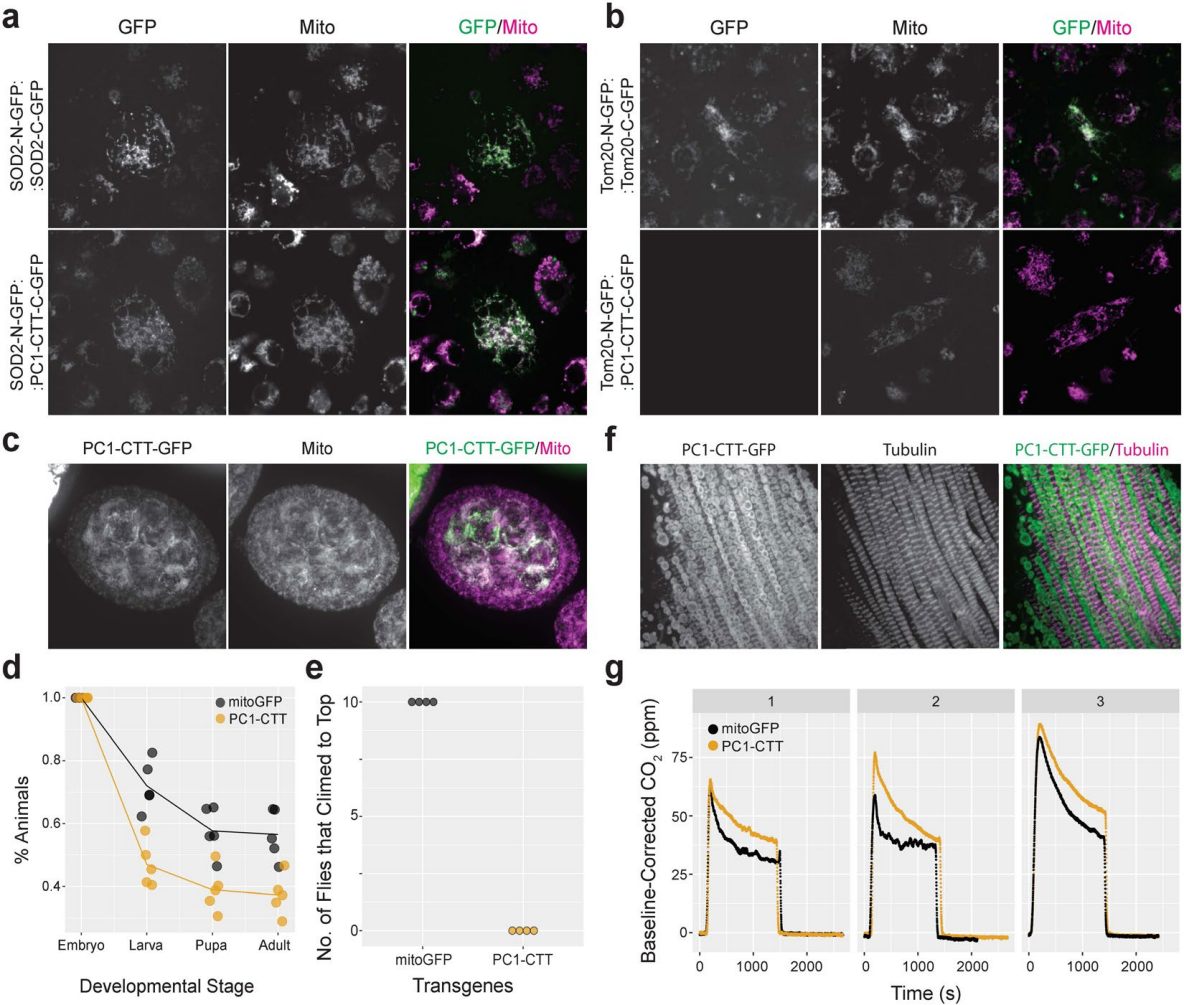
PC1-CTT alters mitochondrial function *in vivo* in *Drosophila*. (**a** and **b**) Split GFP assay showing PC1-CTT-C-GFP complements SOD2-N-GFP in the mitochondrial matrix (**a**) but not Tom20-N-GFP on the mitochondrial outer membrane (**b**). SOD2-N-GFP:SOD2-C-GFP and TOM20-N-GFP:TOM20-C-GFP show complementation and serve as positive controls. (**c**) PC1-CTT-GFP localizes to mitochondria in *Drosophila* ovary (*nanos-gal4* > *UASp-PC1-CTT*). (**d**) PC1-CTT expression in Da-Gal4 flies was associated with a 22% drop in the number of embryos that hatched into first instar larvae (n = 5 experiments; mitoGFP: 419-474 embryos/experiment; PC1-CTT: 317-347 embryos/experiment, p = 0.04). (**e**) Flies expressing PC1-CTT (*Mef2-gal4* > *UASp-PC1-CTT*) in somatic muscle show negative geotaxis climbing impairment compared with control (*Mef2-gal4* > *UASp-mitoGFP*). (**f**) Flies expressing PC1-CTT (*Mef2-gal4* > *UASp-PC1-CTT*) show no obvious morphological abnormalities in muscle cells [green: PC1-CTT in mitochondria; magenta: tubulin]. (**g**) Flies expressing PC1-CTT (*Mef2-gal4* > *UASp-PC1-CTT*) have increased CO_2_ production compared with control (*Mef2-gal4* > *UASp-mitoGFP*). Each panel is one independent experiment showing the baseline-subtracted CO_2_ in 20 flies/group in approximately 30 min.

We used a split GFP assay^30^ to investigate if PC1-CTT localizes to mitochondrial outer membrane or matrix. In insect cells co-expressing PC1-CTT fused to the C-terminal half of GFP with either SOD2 (a mitochondrial matrix protein) or Tom20 (an outer mitochondrial membrane protein) fused with the remaining N-terminal half of GFP, GFP function is reconstituted when both GFP halves are in close proximity (i.e. in the same compartment). Using this assay, we found that GFP function was reconstituted with SOD2-N-GFP, demonstrating that PC1-CTT is present in the mitochondrial matrix (Fig. 8a and b).

To identify *in vivo* effects of PC1-CTT, we selected a tractable system with ease of transgenesis. We therefore generated transgenic fly lines (*Drosophila melanogaster*) expressing either eGFP with a mitochondrial targeting sequence (mitoGFP^31^) or PC1-CTT-eGFP (the last 170 amino acids of mouse PC1: mPC1-4119-eGFP; Fig. 5c). *In vivo*, we find that PC1-CTT also traffics to mitochondria (Fig. 8c). Whole-animal fitness was assayed by analyzing the number of fly eggs that hatched and developed into adults when transgene expression was ubiquitously expressed in embryonic stages under the control of Da-Gal4. Compared to flies expressing mitoGFP, expression of PC1-CTT was associated with a 22% drop in the number of embryos that hatched into larvae (n = 5 experiments; mitoGFP: 419-474 embryos/experiment; PC1-CTT: 317–347 embryos/experiment, p = 0.04; Fig. 8d). We next looked at the effect of PC1-CTT in endurance exercise as a measure of mitochondrial fitness. mitoGFP and PC1-CTT expression were driven in somatic muscle using Mef2-Gal4 and a negative geotaxis climbing assay measured how many flies expressing each of the transgenes could climb 15 cm in 1 minute. All control flies reached the top in the allocated time, contrasted with none in PC1-CTT (n = 4 experiments, total 80 flies, p < 0.001; Fig. 8e). No obvious morphological muscle abnormalities were observed (Fig. 8f), and mitochondrial biogenesis as assayed by measurement of mitochondrial biomass was unaltered (Supplementary Fig. S7). To correlate the endurance test with mitochondrial function, we assayed whole animal respiration and observed consistently higher CO_2_ production in flies expressing PC1-CTT (n = 3 experiments, each with 20 flies/group; p < 0.001; Fig. 8g). These results therefore suggest that heterologous expression of PC1-CTT in *Drosophila* can affect mitochondrial function *in vivo*.

## Discussion

The complete *Pkd1* gene sequence has been available for over 20 years and yet we remain uncertain of its direct, primary function. It has been postulated to serve as a sensor for cell-cell adhesions, cell-matrix interactions, luminal flow or possibly other molecular signals, in keeping with the different subcellular regions where the protein has been reported to localize. A recent study suggests that its activity is modulated by oxygen sensing pathways and that PC1 may indirectly affect mitochondrial function by enhancing their calcium uptake^21^. Our findings suggest a novel, primary function for PC1 directly within the mitochondria.

These data add to a growing body of evidence that suggests a link between PC1 function and cellular metabolism. For example, interventions that target metabolic systems have shown benefit in *Pkd1* mutant mice^23,32,33^. We found through network studies of gene expression patterns of young mice that metabolic pathways were important factors in the critical switch that defines the kinetics of cyst formation after *Pkd1* inactivation^18^. This study also showed that disease-specific gene sets were enriched for metabolic genes, and HNF4α was identified as a key network node that provides partial protection^18^. Metabolic genes were also dysregulated in adult onset *Pkd1* disease models, and mutant kidneys had distinct metabolites and complex lipid profiles^19,20,23^. Several studies have reported cell-autonomous defects in glycolysis and fatty acid oxidation in *Pkd1* knock-out cells, suggesting an intrinsic cellular metabolic dysfunction^19–22^.

Multiple other lines of evidence suggest that dysregulated cellular metabolism is a common feature of many forms of PKD. Knocking-out liver kinase 1 (*Lkb1*), a regulator of glucose and lipid metabolism anecdotally linked to kidney cysts in humans^34^, resulted in tubulointerstitial kidney damage with fibrosis, cysts, and altered mitochondrial morphology in mice^35^. There also is an emerging appreciation of the relationship between ciliary function and regulation of cellular metabolism on both a genetic (ANKS6, NPHP9) and functional level^36–39^. More direct links between mitochondrial dysfunction and cystogenesis are suspected in glutaric academia type II (OMIM 231680), caused by mutations that disrupt electron transfer (ETFA, ETFB, ETFDH^40,41^) and in fumarase deficiency (OMIM 150800)^42,43^. More recently, NADH dehydrogenase (complex I), another electron transfer enzyme, was shown to interact with Fat (Ft) cadherin in *Drosophila*^44^. Ft, a cell adhesion protein, releases a cleavage product that is imported into mitochondria, where it regulates complex I activity. This cross-talk between Ft and complex I affects planar cell polarity^44^, a pathway implicated in the regulation of kidney tubule diameter^45^.

Our current work extends these findings and suggests a direct link between PKD proteins and control of mitochondrial activity. We show that *Pkd1* knockout cells have different metabolic fluxes with likely altered oxidoreductase activity, consistent with changes in NAD+/NADH. Together with the changes in acetyl-CoA isomers and fatty acid accumulation in uptake assays, our results are consistent with impaired fatty acid oxidation^20^. Furthermore, in *Pkd1* knockout cell lines, we show evidence of altered mitochondrial membrane potential and abnormal mitochondrial networks, and in cell lines and kidneys of *Pkd1* knockout mice and in ADPKD patient samples, we show ultrastructurally abnormal mitochondria, characterized by smaller, swollen mitochondria, with less evident cristae.

We have identified a novel, functionally conserved MTS that is capable of trafficking GFP to the mitochondria in species ranging from humans to *Drosophila*. We have shown that the PC1 CTT cleavage product traffics to mitochondria in different types of cells from multiple species. We confirmed microscopy studies using cell fractionation and biochemical methods. In addition, we used MEFs expressing an HA-tagged BAC to verify CTT enrichment in mitochondrial fractions using a surrogate model of endogenous PC1 expression (ie. MEFs from a mouse line with an HA-tagged *Pkd1* BAC transgene shown to functionally rescue *Pkd1* mutants^25^).

We have also shown that re-expression of PC1 CTT in mutant *Pkd1* cell lines correlates with increased mitochondria elongation, partially rescuing the mitochondrial fragmentation phenotype. Mitochondria are notoriously dynamic, constantly moving around and undergoing fusion/fission^46^. In *Pkd1* mutant cells, our data suggest that the mitochondrial network is still dynamically changing: in some cells mitochondria are highly fragmented; in others, elongated. In fact, this dynamism is one likely cause for the variability of the solidity index shown in Fig. 2f. Despite the dynamic nature of this process, recombinant PC1 CTT can consistently nudge the network structure towards more elongated structures (Fig. 7). Whether this is accomplished by direct regulation of fusion/fission machinery, or through indirect metabolic cues, is an interesting question for future investigation.

Our fly data suggest that PC1 CTT expression might also affect mitochondrial function *in vivo*. While flies have a homologue of PC1, its expression is restricted to fly sperm and it lacks much of the CTF including the last transmembrane domains and the CTT. Nonetheless, we reasoned that if PC1 CTT interacts with mitochondrial proteins, many of which are highly conserved, expressing PC1 CTT in flies might disrupt mitochondrial function. We therefore scored phenotypes commonly associated with mitochondrial disease: early embryonic lethality when the expression was ubiquitous^31,47^, or endurance to exercise and metabolic rate (CO_2_ production) when expression was restricted to muscle^47,48^. We showed that transgenic over-expression of PC1-CTT in flies results in higher embryonic lethality, lower exercise endurance and increased CO_2_ production, consistent with our prediction. While reduced exercise endurance and increased CO_2_ production might seem to be paradoxical effects, both phenomena could result if PC1-CTT causes mitochondrial uncoupling in the muscles of transgenic flies. It is also possible, however, that the two observations result from different processes. Mef2 is expressed in adult neurons involved in the regulation of circadian rhythm as well as in muscle cells so PC1-CTT might also affect mitochondrial function in that cell type. Given the known relationship between circadian rhythm and metabolic homeostasis, we cannot exclude such an effect in our fly model. It is worth noting, however, that regardless of the cell type whose dysfunction is ultimately responsible for the observed effects, the fact that there are multiple measureable abnormalities strongly suggests that expression of recombinant CTT-GFP in flies affects mitochondrial function *in vivo*. Further study will be required to identify the underlying mechanisms.

The observation that expression of PC1 CTT can both rescue a mutant phenotype and cause mitochondrial dysfunction in an otherwise normal system is an interesting paradox. While this might seem inconsistent, it should be noted that there are ample examples of conditions where either recessive mutations (loss of function) or dominant (gain of function/dominant negative) mutations of the same gene result in disease affecting the same system^49–51^. One potential model to explain these observations is that PC1 has an essential function in vertebrate cells that normally express it so its loss results in cellular dysfunction that can be partially rescued by its re-expression. In Drosophila, which normally lack PC1 CTT, the transgenic protein instead interferes with the normal function of a mitochondrial partner, possibly by competing with an endogenous protein.

Our findings have important differences with published reports^7,8,21^. Two groups have previously described a nuclear localization pattern for the CTT and in each case the size was distinct (34kDa^7^ and 17 kDa^8^). While it is theoretically possible that the longer product described by Chauvet *et al*. has a nuclear translocation signal that is missing from our shorter constructs (~17kDa), this is unlikely to explain the differences, since Low *et al*. had concluded that nuclear localization was mediated by the C-terminal half of the PC1 tail. This also does not explain why none of our full-length constructs tagged at their C-termini with either fluorescent proteins, FLAG or HA epitopes yielded a nuclear pattern. One possible explanation for these differences is that, under standard conditions, HA-tagged PC1-CTT is harder to detect. In fact, we initially only detected it when constructs were tagged with fluorescent proteins. Even with the short constructs that mimic those used by Low *et al*., non-GFP, single-tagged HA constructs were detectable in the mitochondria only after we had optimized permeabilization conditions using doubly tagged (ie. HA and eGFP) CTT constructs.

PC1 also has been localized to mitochondria associated membranes (MAMs), points of contact between the endoplasmic reticulum and mitochondria. While this certainly is plausible given the relative abundance of PC1 in the ER, we note that localization of PC1 to MAMs was done solely using a biochemical marker that maps to a number of locations within the cell in a cell-type dependent manner (Protein Atlas http://www.proteinatlas.org/ENSG00000068366-ACSL4/cell#rna) and peroxisomes^52^. We also cannot exclude the possibility that the CTF and other C-terminal products, which were variably enriched in mitochondria fractions, localize to these structures. Immunoelectron microscopy may help to unambiguously resolve this issue. Finally, we did not see that hypoxia or interventions known to mimic hypoxia had any effects on the mitochondrial pattern, but we did not examine PC1 patterns in conditions where the cells are ciliated.

Our study has several limitations. We have not yet been able to show localization to mitochondria by microscopy of endogenous PC1-CTT. Because PC1 is expressed at very low levels in most tissues and cells and not reliably detectable by immunofluorescence, for now we must rely on recombinant systems. We also have made extensive use of cell lines rather than “normal” primary cells. The very large size of the cDNA (>13 kb) makes transient expression in primary cells very difficult as the construct is too large for standard viral delivery systems and conventional methods have a very low level of efficiency for something this size. Therefore, we cannot yet conclude definitively that endogenous PC1 has the same functional properties as our recombinant protein. We also have thus far only shown partial rescue of mitochondria fragmentation with the PC1-CTT and thus do not yet know whether it can correct other mitochondrial abnormalities in *Pkd1* mutants. Finally, our *in vivo* functional studies have thus far been limited to ectopic expression of PC1-CTT in *Drosophila;* thus, it is still unknown if mitochondrial PC1-CTT plays any physiological role in vertebrates.

Collectively, both our new data and other recent findings prompt consideration of the broader question of how cellular metabolism, mitochondrial activity and cellular morphology/tissue structure are interconnected. Cellular metabolism is known to respond and sometimes to determine cell fate and differentiation (reviewed in^53^). In muscle cells, for instance, mitochondria are highly attuned to tissue properties, suiting their metabolic profiles to changes in muscle fiber type^54^. In cancer, too, metabolic reprogramming is thought to be important, involving not only a shift towards glycolysis (the Warburg phenomenon), but also engaging mitochondria as a source of building blocks for biosynthesis (reviewed in ref.^55^). Mitochondria also may relay information through modulation of acetyl-CoA/CoA ratios and histone acetylation^56^, thereby allowing rates of FAO to orchestrate global changes, as shown for lymphangiogenesis^57^. Mitochondria also appear to be an important downstream target of signaling systems linked to cell-cell adhesions. One cell-cell adhesion regulatory protein (CCARP) identified in a genome-wide screen, ADCK4, interacts exclusively with mitochondrial proteins^58^. Multiple other proteins were also found to interact with both known cell-cell adhesion pathway targets and mitochondrial proteins.

**Figure 9:**
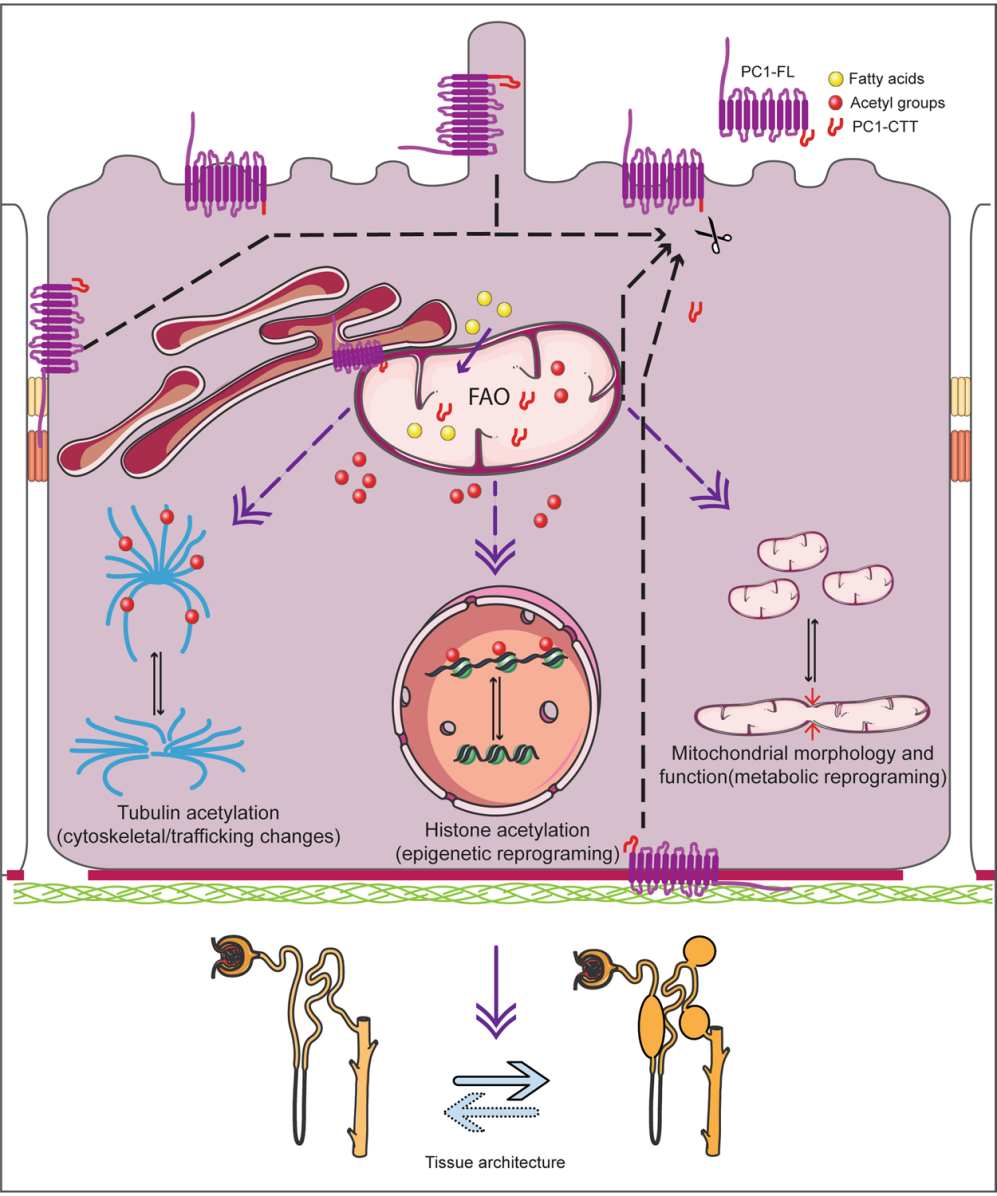
Model of how PC1 signaling could alter nephron architecture. PC1 could respond to ligand binding or mechanical stimuli (from cilia, cell-cell/matrix) by modulating the amount of PC1-CTT that is released and traffics to mitochondria. In mitochondria, PC1-CTT could impact fatty acid oxidation (FAO) either by regulating fatty acid uptake or mitochondrial function. Altered FAO could change the pool of acetyl-CoA (and possibly ROS and NAD+/NADH), with effects in tubulin acetylation (affecting trafficking and cytoskeleton) and histone acetylation (possibly with global gene expression reprogramming), both previously linked to PKD^77,78^. Alternatively, PC1-CTT may directly change mitochondrial fusion/fission rates, changing the mitochondrial network and function. How these changes would result in cystic change is not clear, but as discussed in the text, a similar cascade was observed in lymphangiogenesis and planar cell polarity, processes previously linked to *Pkd1* function^45,79,80^. This model does not include PC2 or regulation of NTF/CTF dissociation, aspects likely relevant, but not investigated in this study.

PC1 may similarly link cell-cell and/or cell-matrix signaling to mitochondrial activity. One model is that PC1 detects mechanical signals and relays information to mitochondria through cleavage/release of CTT, which fine tunes fatty acid transport/oxidation and affects mitochondrial membrane properties. Effects on acetyl-CoA levels may mediate epigenetic or cytoskeletal changes^59^ which result in metabolic reprogramming and altered ROS signaling. These processes ultimately regulate cell shape and tissue architecture (Fig. 9). In conclusion, our data may provide a unifying explanation and framework for investigating the link between PKD and cellular metabolism.

Note:While this manuscript was under revision, Ishimoto *et al*. reported similar mitochondrial phenotypes in ADPKD patients and models^60^.

### Ethics Statement

All studies were performed using protocols approved by NIH Animal Care and Use Committee or by The University of Maryland School of Medicine Institutional Animal Care and Use Committee, as appropriate. Mice were kept and cared in pathogen-free animal facilities accredited by the American Association for the Accreditation of Laboratory Animal Care and meet federal (NIH) guidelines for the humane and appropriate care of laboratory animal. The human specimens were collected with informed consent under an IRB-approved protocol at the Juntendo University Graduate School of Medicine (#17-032).

## Methods

### Cell lines

Flp-In tetracycline inducible (ThermoFisher Scientific) MDCK cell lines were previously described^61^. Additional cell lines expressing N-terminal eGFP- and C-terminal HA-tagged human PC1 and pcDNA5 vector control were generated following the manufacturer’s instructions. Cells were cultured in Dulbecco’s minimal essential medium (DMEM) supplemented with 10% fetal bovine serum (FBS) with 1% penicillin/streptomycin and selection agents 100 *μ*g/ml hygromycin B and 5 *μ*g/ml blasticidin. PC1 expression was induced with 10 *μ*g/ml tetracycline.

Kidney cell lines derived from *Pkd1*^*cko/cko*^ animals were immortalized and inactivated *in vitro* as previously described^20^. Briefly, kidneys from several mice (121112-C: male, <12-days-old; 94414: male, 463-days-old; 96784: male, 61-days-old) were harvested, minced and digested using a collagenase/hyaluronidase solution (Stemcell technologies, cat. no. 07912) followed by proximal or collecting/distal tubule cell enrichment using, respectively, biotinylated Lotus Tetragonolobus Lectin (LTL) (Vector Laboratories, cat. no. B-1325) or biotinylated Dolichos Biflorus Agglutinin (DBA) (Vector Laboratories, ca. no. B-1035) and Cellection Biotin Binder kit (ThermoFischer, cat. no. 11533D). Cells were immortalized using the large T antigen (Addgene plasmid no. 22298; a gift from Eric Campeau) and *Pkd1* was conditionally inactivated using cre recombinase (121112C-LTL cells; Excellgen, cat. no. EG-1001) or viral transduction (121112-C DBA and 94414-LTL/DBA cells) using LV-Cre (Addgene 12106^62^). At the time of inactivation, a corresponding control was generated using viral transduction with plasmid LV-Lac (Addgene 12108^62^). *Pkd1* inactivation was confirmed using genomic PCR and/or reverse-transcriptase PCR (TaqMan gene expression assay, Applied Biosystems, cat. no. 4351372, Mm00465436_g1). Cells were grown in DMEM/F12 media (Life cat. no. 21041-025) with 2% FBS (GEMINI Bio-Products cat. no. 100-106), 1 × Insulin-Transferrin-Selenium (Thermo Fisher Scientific, cat. no. 41400-045), 5 µM dexamethasone (SIGMA, cat. no. D1756), 10 ng/ml EGF (SIGMA, cat. no. SRP3196), 1 nM 3,3′,5-Triiodo-L-thyronine (SIGMA, cat. no. T6397) and 10 mM HEPES (CORNING, cat. no. 25-060-CI).

Mouse embryonic fibroblasts (MEFs) were obtained from E12.5 wild type or *Pkd1*^*F/H*^ BAC mice (expressing mouse *Pkd1* with three C-terminal HA tags^25^). Whole embryos were minced, washed in PBS and cultured in six-well tissue culture plates in Dulbecco’s minimal essential medium (DMEM) supplemented with 10% fetal bovine serum (FBS).

### Constructs

A plasmid for transient expression of full-length human PC1 was modified from the Addgene plasmid #21359^4^ using a combination of restriction digestion, gBlocks gene fragments (Integrated DNA Technologies) and In-Fusion HD cloning (Clontech). The resulting AF20-double-tag plasmid expresses the full-length human PC1 with N-terminal mCherry-TEV cleavage-V5 and C-terminal –eGFP-TEV cleavage-S15 tags. A pcDNA5-backbone plasmid for stable expression in Flp-In cells was modified from^61^ and expresses full-length human PC1 with N-terminal eGFP and C-terminal HA tags. Constructs expressing PC1-CTT with either eGFP, eGFP-3HA or 3HA C-terminal tags were cloned into pcDNA3 and include the following PC1 sequences: PC1 insert amplified from pcDNA4/TO CD16.7-h-PKD1 FLM (4078-4303) (Addgene plasmid #83465^63^); and the following mouse PC1-CTT inserts with the addition of 5′ Kozak sequences: PC1-CTT (mPC-4119): ACCatggtggagcttttcctgcgtaggcttcggctctggatgggcttcagcaaggtcaaggagttccgccacaaagtccgctttgaaggaatggatccactgccttcccgctcatccaggggctccaagtcatccccagttgtgctcccacctagctcaggctcagaagcttcacacccatccacctcgtccagccaaccagacgggccgagcgccagcttaagccgctcgacgctgaagctggaaccagagccctctcgcctccatgctgtgtttgaaagtctgcttgtccagtttgaccgactcaaccaggccacagaggacgtctaccagctggagcaacaactccagagccttcaaggccatgggcacaatggacctccttcctctccctcccctggctgcttcccaggctctcagccagctttgcccagccgcctttctcgggccagtcaggggctggatcagactgtaggccccaacagggtgtccctgtggcctaataacaaggtccaccccagcagcact; and a truncated mPC1-4148 lacking the putative mitochondrial targeting sequence (MVELFLRRLRLWMGLSKVKEFRHKVRFEG): ACGATGatggatccactgccttcccgctcatccaggggctccaagtcatccccagttgtgctcccacctagctcaggctcagaagcttcacacccatccacctcgtccagccaaccagacgggccgagcgccagcttaagccgctcgacgctgaagctggaaccagagccctctcgcctccatgctgtgtttgaaagtctgcttgtccagtttgaccgactcaaccaggccacagaggacgtctaccagctggagcaacaactccagagccttcaaggccatgggcacaatggacctccttcctctccctcccctggctgcttcccaggctctcagccagctttgcccagccgcctttctcgggccagtcaggggctggatcagactgtaggccccaacagggtgtccctgtggcctaataacaaggtccaccccagcagcact. MTS-eGFP constructs were generated using gBlocks (Integrated DNA Technologies) and included the MTS sequence (or variants) fused to eGFP. The gBlocks were cloned into pcDNA3 using In-Fusion HD (Clontech).

For split-GFP assays, constructs were cloned into a polycistronic split GFP vector derived from Ac4-Stable 2-neo (Addgene # 32426^64^), as described previously^30^.

### Fluxomics

Confluent dishes (three technical replicates) of 94414-LTL control and knock-out were rinsed in PBS with1mM EGTA, switched to fresh media for 30 min. and treated with DMEM with 2 mM glutamine, 100 mM oleate, and either 5 mM ^13^C-glucose (Sigma, 389374) or 5 mM unlabeled glucose for 3 h. The cells were then rinsed in 10 ml of 150 mM ammonium acetate and frozen in liquid nitrogen. ^13^C mass isotopomer analysis using GC-MS was performed by the Michigan Regional Comprehensive Metabolomics Resource Core, at the University of Michigan, following the core’s protocols. The experiment was performed once. The relative fraction of mass isotopomers for each metabolite was used for analysis.

### Fatty acid uptake assay

Genetically matched mutant/control pairs of mouse kidney epithelial cells were grown to confluency, then treated with 100 μM of Palmitic Acid (Cambridge Isotope Laboratories, Inc. DLM-215-1) and 10 μM of BODIPY® 558/568 C_12_ (Thermo Fisher Scientific Inc. Catalog number: D38354, 4-Difluoro-5-(2-Thienyl)-4-Bora-3a,4a-Diaza-*s*-Indacene-3-Dodecanoic Acid) overnight. Cells were washed with PBS then stained with 100 nM of MitoTracker® Green FM (Thermo Fisher Scientific Inc. Catalog number: M751) for 30 min. Images were randomly taken by ZEISS LSM 700 using the Plan-Apochromat 63 ×/1.40 Oil DIC M27 lens. Quantification of lipid droplet size and number was done in 10 randomly selected fields in two cell lines using ImageJ. An investigator blinded to genotype adjusted image intensity thresholds to detect only lipid droplets prior to the analysis. The experiment was performed four times.

### TMRM assay

We used flow cytometry to assess mitochondrial polarization. Briefly, we re-suspended 1 × 10^6^ cells per ml of PBS in presence or absence of 25 nM TMRM indicator (#T668; Thermo Scientfic Inc) and/or 10 μM CCCP (C-2759; Sigma) for 20 min. at 37 °C. Then the cells were washed two times with PBS and re-suspended in 500 μl fresh PBS. At this stage cells were kept on ice and were analyzed using Flow cytometry. Samples were acquired on LSRFortessa (BD, San Jose, USA) equipped with 355 nM, 407 nM, 488 nM, 532 nM and 633 nM LASER lines using FACSDIVA software. Data was recorded upon excitation with 532 nM LASER line and emission was collected using 575/25 bandpass filters in log scale. 50,000 single live cells were recorded to quantify the degree of mitochondrial polarization. TMRM intensity was corrected for cell size and granularity using a linear fit, as previously described^65^. The experiment was performed at least three independent times for each of three independent cell lines.

### Immunofluorescence microscopy

Cells were seeded on coverslips (Poly-D-lysine coated German coverslip for cell culture, Neuvitro Corporation H-22-1.5-pdl) in 6-well tissue-culture-treated plates the day before transfection. Cells were transfected with pcDNA3-mPC1-4119-3HA or pcDNA3-mPC1-4119-eGFP-3HA by FuGENE® 6 Transfection Reagent (Promega Corporation E2691) following manufacturer’s instructions. For protease inhibition, cells were incubated with 30 μM of MG-132 (Sigma C2211, Synonym: Z-Leu-Leu-Leu-al) for 3 h before fixing in 10% Neutral Buffered Formalin (VWR 16004-128) for 10 min. Cells were then permeabilized in 0.5% Triton X-100 in PBS for 10 min., blocked in 10% fetal bovine serum in PBS for 30 min., and incubated for 1 h at room temperature with primary antibodies against HA (mouse monoclonal, 1:100; MBL International M180-3). After washing with PBS for three times, cells were incubated in darkness with secondary antibody (Donkey anti-Mouse IgG (H + L) Highly Cross-Adsorbed Secondary Antibody, Alexa Fluor 647 1:1000; Thermo Fisher Scientific Inc A-31571) for 30 min. and mounted with mounting fluid (VECTASHIELD HardSet Antifade Mounting Medium, Vector Laboratories H-1400). Images were captured with a Zeiss LSM700 Confocal microscope using the Plan-Apochromat 63 ×/1.40 Oil DIC M27 lens.

### Live cell microscopy

Cells were grown in a 35 mm µ-Dish with #1.5 polymer coverslip (ibidi, cat. no. 81156) the day before transfection. Cells were transfected with plasmids expressing either PC1-CTT or mCherry-PKD1-eGFP, mito-BFP (Addgene 49151) for mitochondrial marker, or pEF.myc.ER-E2-Crimson for ER marker (Addgene 38770^66^) by FuGENE® 6 Transfection Reagent (Promega Corporation E2691) following manufacturer’s instructions. For counterstaining of mitochondria, cells were incubated in 100 nM of MitoTracker® Deep Red FM (Thermo Fisher Scientific M22426) or MitoTracker® Green FM (Thermo Fisher Scientific M7514) for 30 min. For ER counterstaining, cells were incubated in 100 nM of ER-Tracker™ Blue-White DPX for 30 min. Images were captured with a Zeiss LSM700, LSM780 or LSM 880 with Airyscan confocal microscope using the Plan-Apochromat 63 ×/1.40 Oil DIC M27 lens. For mitochondrial network rescue experiments, *Pkd1*^*ko/ko*^ renal epithelial cells in suspension were transfected with plasmids expressing either hPC1-CTT-eGFP or hPC1-MTS-eGFP by FuGENE® HD Transfection Reagent (Promega Corporation E2311) the day before assay. Images were acquired with a Zeiss LSM780 using the Plan-Apochromat 63 ×/1.40 Oil DIC M27 lens. A total of two to four independent experiments analyzing three independent mutant cell lines were used.

### Mitochondrial network quantification

For quantification of mitochondrial network structure, an average of 50 images of random fields of cells stained with mitotracker were imaged. A total of 13 independent experiments analyzing three cell lines with matched mutant/control samples were used. Identification of mitochondria and determination of shape parameter was done using CellProfiler^67^ using a pipeline modified from^68^. As a measure of network fragmentation we used the solidity index, which had been previously reported to distinguish fission/fusion events in mitochondria (i.e. fragmented/connected mitochondrial networks)^24^. Briefly, solidity measures the complexity/spikiness of a shape by dividing the area of the shape by the area of the smallest convex polygon that encloses the shape (i.e. the solidity of a star-shape object would be the area of the star divided by the area of the smallest circle around it; so, for a star with narrow rays, solidity would be low but would be higher if the star has broad rays). A network of connected mitochondria has low solidity whereas fragmented, disconnected mitochondria have higher values (Supplementary Fig. S2).

### Electron microscopy

Electron microscopy (EM) of mouse kidneys and cultured mammalian cells was performed as described previously with minor modifications^69^. Briefly, 94414-LTL mutant and control cells were cultured to 80% confluence, fixed in 3% paraformaldehyde in 0.1 M sodium cacodylate buffer (pH 7.4) for 15 min at room temperature, washed with 0.1 M sodium cacodylate buffer (pH 7.4) once, scraped off the culture and post-fixed and processed as below. Kidneys from 7-month old female fifth-generation C57/BL6 *Pkd1*^*cko/cko*^ ^70^ positive or negative (control) for a tamoxifen-inducible Cre transgene (B6.Cg-Tg(Cre/Esr1)5Amc/J mice (stock 004682), Jackson Laboratories) mice, treated with tamoxifen at P40 as described^20^, were harvested, minced into 0.5 mm^3^ fragments, dissociated in 1 × collagenase/hyaluronidase solution (STEMCELL technologies, cat. no. 07912) with 1 U/ml dispase (STEMCELL technologies, cat. no. 07923), washed in PBS and fixed in a mixture of 2.5% glutaraldehyde, 1.25% paraformaldehyde, and 0.1 M sodium cacodylate buffer (pH 7.4) for 1 hr on ice, washed twice in 0.1 M sodium cacodylate buffer, postfixed with 1% Osmium tetroxide in 0.1 M sodium cacodylate buffer for 1 hr, and washed twice in distilled water. To enhance contrast, fixed cells were stained with 2% aqueous uranyl acetate for 1 hr at room temperature. Samples were then dehydrated in increasing concentration of ethanol (50%, 70%, 80%, 90%, 95%, and 100%) for 10 min. each, with two more incubations in 100% ethanol from a freshly opened bottle. Samples were rinsed twice in acetone (EM grade), infiltrated and embedded in Embed 812 resin (EMS, Hatfield, PA, USA) for 1 hr each with a 3:1, 1:1, 1:3 acetone/embedding media mixture. Cells were incubated overnight with 100% fresh resin. The next day, cells were again re-suspended in fresh 100% resin for 2-3 hrs, transferred into BEEM capsules (EMS, Hatfield, PA, USA), and polymerized at 65 °C for 48 hr. Semi and ultrathin sections were produced with a diamond knife (Diatome, Biel, Switzerland) on an ultra-microtome (Ultracut UCT, Leica-Microsystems), collected on formvar coated 200 mesh copper grids (EMS, Hatfield, PA, USA), post stained with uranyl acetate and lead citrate, and examined with a Tecnai T12 TEM (FEI), operating at 120 kV. Images were recorded on a below mounted Gatan 2k × 2k CCD camera. Brightness and contrast were adjusted using Adobe Photoshop CC14. One to two images were taken per cell and used for the analyses.

Human kidney biopsies were obtained at the time of kidney transplant from two patients and processed as above, with the following modifications. Samples were fixed in modified Karnovsky solution (of 2% glutaraldehyde and 2% paraformaldehyde in 0.1 M sodium phosphate buffer, pH 7.4) and examined with a JEM-1011 TEM (JEOL). Patient 1: 59 year-old male, undergoing dialysis for 46 months prior to kidney transplantation Patient 2: 66 year-old male, with 4.93 mg/dl creatinine at the time of transplant, and with a family history of ADPKD (an older brother).

### Immunoblots and immunoprecipitation

Cells were lysed in lysis buffer (25 mM Sodium Phosphate (pH 7.2), 150 mM NaCl, 10% Glycerol, 1 mM EDTA, 1% TritonX-100) and Complete protease inhibitor mixture (Sigma, 4693116001). Samples were electrophoresed on 4-12% gradient SDS-PAGE gels after reducing with dithiothreitol-based reducing agents (Thermo Scientific Inc., NP0009). Western blotting was performed with an iBlot Gel Transfer device and nitrocellulose membrane (Thermo Scientific Inc., IB301002). Chicken polyclonal anti-GFP antibody (Aves, GFP-1020) and mouse monoclonal anti-HA (MBL, M180-3) were used for probing. Bands were detected and quantified using an Odyssey Infrared Imaging System (LI-COR). In some experiments, band intensities of PC1 fragments were normalized to PC1 full-length band intensity.

Mitochondrial isolation was performed (n = 7 independent experiments) using Qproteome Mitochondrial Isolation Kit (QIAGEN, 37612) according to manufacturer’s manual. Sub-fractional enrichment was confirmed by probing with anti-Timm23 (BD bioscience, 611222) and anti-Heterochromatin protein-1β (Millipore, MAB3448) antibody.

For immunoprecipitation, cell lysates from MDCK and MEF were incubated with mouse monoclonal anti-HA antibody (MBL, M180-3) and Protein G agarose (Sigma, 11719416001). MDCK cells with tetracycline-inducible stable PC1 expression (with GFP tag on its N-terminus and HA tag on its C-terminus) and control were induced with 10 *μ*g/ml tetracycline for 48 h prior to studies. Cells were exposed to: 1) hypoxia (0.01%O_2_, 5%CO_2_) for 2 hours (n = 3 experiments); 2) treatment of 1 *μ*M CCCP (Carbonyl cyanide 3-chorophenylhydrazone; Sigma, Aldrich, St. Louis MO) for 20 min. followed by 16 hour incubation in standard medium (n = 3 experiments); 3) starved for 48 h in serum-free DMEM (n = 1); 4) 24 h treatment with “inhibitors mix” (n = 2 experiments): protease inhibitor cocktail (1:200 P1860; Sigma), calpain inhibitor (26 μM ALLN, SantaCruz, sc-221236), matrix metalloproteinase inhibitor (6 pg/μl Batimastat; ApexBio, A2577); 5) 3 h treatment with proteasome inhibitor (20 μM MG132, C2211, Sigma; n = 2 experiments); 6) 24 h γ-secretase inhibitor (50 μM DAPT, D5942, Sigma; n = 3 experiments). Band intensity was measured using Fiji^71^, and normalized to PC1-FL band intensity. Rabbit polyclonal anti HIF-1 alpha Antibody (Novus Biologicals, NB100-479) was used to confirm Hif1α induction in the total lysate.

### Fly experiments

#### Genetics and maintenance

All *D*. *melanogaster* strains were maintained on standard corn meal, agar, molasses medium at 25 °C.Transgenic flies expressing PC1-CTT-eGFP were generated through standard P-element transformation carried out by BestGene Inc. mitoGFP flies were described previously^31^ and express N-terminal 40 amino acid residues of *Drosophila* citrate synthase (CG3861), as a mitochondrial targeting sequence, fused to GFP. All fly stocks were obtained from Bloomington Drosophila Stock Center (Bloomington, IN).

#### Split GFP assay

This assay was performed as previously described^30^. Briefly, a fusion protein consisting of mouse PC1-CTT and the C-terminal half of GFP was co-expressed with a known mitochondrial protein fused with the N-terminal half of GFP. If two fusion proteins localize within the same sub-compartment, two half GFPs will complement and reconstitute into a functional GFP. Tom20 and SOD2 were used as markers for outer mitochondrial membrane and mitochondrial matrix, respectively.

#### Fly viability analysis

Homozygous females from the UAS-mitoGFP and UAS-PC1-CTT lines were crossed with homozygous males from Da-Gal4 line at 25 °C. The viability assay was replicated five times and was done in vials with one virgin female and three virgin 2–5 days old males. Flies were flipped into new vials every day for eight days. 24 h after the flies were removed from a vial, the number of unhatched eggs and larvae were counted. The numbers of pupae and emerging adult flies were also recorded. The experiment was performed five times.

#### Climbing assay

Ten flies were randomly picked and were individually transferred into a long glass tube by tapping down from the original vials. The predefined finish height was 15 cm. The time for each fly to climb up was 60 s. The number of flies to climb to a 15-cm line in 60 s was recorded. Four groups of 10 flies for each genotype of 2–5 days old were tested. The experiment was performed four times.

### CO_2_ production assay

CO_2_ production in an open flow system was monitored for 30 min. at room temperature using a Q-Box RP1LP low-range respiratory system (Qubit, Kingston, Canada). The experiment was performed three independent times using twenty 2-3 day-old male flies for each group.

### Statistics

Statistical analysis was performed in R/Bioconductor^72,73^, using the limma^74^ and, for graphs, ggplot2^75^ and ggfortify^76^ packages.

Fluxomics data were analyzed fitting relative fraction of mass isotopomers for each metabolite by genotype followed by empirical bayes estimation of differential expression (eBayes).

In the fatty acid uptake assay, the median lipid droplet area in four independent experiments was compared between mutant and control cells using paired t-test.

Electron microscopy images were compared fitting an analysis of variance model for mitochondrial morphology (proportion of aberrant) and genotype, within cell line (n = 110 fields for 94414-LTL; n = 68 fields for primary cells).

For mitochondrial network comparisons, we used paired t-test comparisons (n = 13 experiments) of the 25^th^ centile solidity in mutant vs. matched control cells. For the mitochondrial network rescue experiments, for each experimental condition the 25^th^ centile solidity of non-transfected was subtracted from transfected cells, producing the data shown in Fig. 7c. Paired t test was used to compare the change in solidity between transfected plasmids for each experiment with each cell line (n = 9).

TMRM fluorescence was analyzed using paired t-test comparing TMRM normalized intensity at the 5^th^ and 95^th^ percentiles for each experimental condition, comparing each cell line to its corresponding control. Similar comparisons at each percentile is shown in Fig. 2c. For Fig. 2b, each percentile for a given cell line/condition/experiment was calculated, and the control value was subtracted from the corresponding mutant TMRM normalized intensity. A value above 0 therefore corresponds to higher TMRM intensity in mutant cells at that quantile.

Fly viability was analyzed by t-test comparing the fraction of first instar larvae per group (n = 5 experiments). The significance of difference in the climbing assay was analyzed using Wilcox test comparing number of flies that climbed to the top in the allotted time. For the CO_2_ respiration, paired t-test (n = 3) compared the mean of baseline-corrected CO_2_ measurements during the time interval where flies (20/measurement) were in the system.

## Electronic supplementary material

Supplementary Table 1

Supplementary Figures
