# Supplementary material for: A cleavage product of Polycystin-1 is a mitochondrial matrix protein that affects mitochondria morphology and function when heterologously expressed
Source: Sci Rep. 2018 Feb 9;8:2743. doi: 10.1038/s41598-018-20856-6 (PMC5807443; doi:10.1038/s41598-018-20856-6)
Supplement: Supplementary file 2 — Supplementary Figures [file 41598_2018_20856_MOESM2_ESM.pdf]

A cleavage product of Polycystin-1 is a mitochondrial matrix protein that affects mitochondria morphology and function when heterologously expressed

Author List: Cheng-Chao Lin[1], Mahiro Kurashige[1], Yi Liu[2], Takeshi Terabayashi[1], Yu Ishimoto[3], Tanchun Wang[1], Vineet Choudhary[4], Ryan Hobbs[1], Li-Ka Liu[4], Ping-Hsien Lee[5], Patricia Outeda[6], Fang Zhou[1], Nicholas P Restifo[5], Terry Watnick[6], Haruna Kawano[7], Shigeo Horie[7], William Prinz[4], Hong Xu[2], Luis F Menezes[1], Gregory G Germino[1].

1. Kidney Disease Branch; National Institute of Diabetes and Digestive and Kidney Disease, National Institutes of Health (NIH), Bethesda, MD
2. Laboratory of Molecular Genetics; National Heart, Lung and Blood Institute, NIH, Bethesda, MD
3. Division of Nephrology and Endocrinology and the Division of CKD Pathophysiology, University of Tokyo Graduate School of Medicine, Tokyo, Japan
4. Laboratory of Cell and Molecular Biology; National Institute of Diabetes and Digestive and Kidney Disease, NIH, Bethesda, MD
5. Center for Cell-Based Therapy, National Cancer Institute, NIH, Bethesda, MD

6. Department of Medicine, Division of Nephrology, University of Maryland School of Medicine, Baltimore, MD

7. Department of Urology, Juntendo University Graduate School of Medicine, Tokyo, Japan

SUPPLEMENTARY LEGENDS

Figure S1. ***Pkd1* wild type and *Pkd1* mutant cells have similar levels of phospholipids.** Comparison of phospholipid composition (as relative abundances of total phospholipids) in several different cell lines (color: genotype; shape: independent experiment)

Figure S2. Schematic illustration explaining how the solidity index is used to assess mitochondrial network structure. Solidity measures the ratio between the area of the shape of interest (schematized mitochondria) and the convex polygon enveloping it (blue polygon). A lower solidity suggests a more networked shape, whereas higher solidity suggest fragmented structures. Content-based image retrieval (CBIR) works on the premise that images can be segmented into shapes that can be measured, described and compared using a set of numeric features [1]. Among the shape-based features, solidity has been singled-out as useful to evaluate mitochondrial morphological features [2] and is defined by:

$$\text{Solidity} = A_s/H$$

where A_s is the area of the shape being evaluated, and H is the area of the convex hull (polygon) surrounding it [1]. As the figure below illustrates, when a shape has branches, the area H of the surrounding polygon (the blue polygon) is bigger than the area A_s of the shape (schematized mitochondria), and solidity is

closer to 0. Simpler, less branched features, will have similar H and A_s, and the solidity will be closer to 1. A fragmented mitochondrial network will therefore have higher solidity than a fused, elongated network.

Figure S3. Immunoblot of mitochondria fraction from MEFs with lower molecular weight markers. Immunoblot showing total cell lysates ("mito –") and mitochondrial enriched fractions ("mito +") of MEFs obtained from wild type or transgenic *Pkd1*$^{F/H}$ BAC mice expressing PKD1-HA. The CTT is ~17-18kDa and is enriched in the mitochondrial fraction. The cropped blots at the bottom of each panel show subsequent staining of the same membrane with nuclear (HP1β) and mitochondrial (Tim23) markers.

Figure S4. *Pkd1*-GFP CRISPR strategy and an informative mis-targeting event.

A) Schematic illustration of the strategy to insert copGFP tag into the *Pkd1* locus. The upper diagram shows the 3' *Pkd1* genomic region, with coding sequences (CDS) shown in black boxes and untranslated regions (UTR) in grey boxes. The position of the single-guide RNA (sgRNA) target is indicated by the arrowhead. The diagram in the middle shows the donor vector with left (5') and right (3') homologous arms (5' ARM and 3' ARM), and the mRuby reporter and puromycin resistance genes driven by the EF1 promoter. The bottom diagram represents the knock-in allele, and the arrows show the location of primers used in PCR reactions to confirm knock-in.

B) PCR reactions of puromycin-resistant clones, showing several clones with amplification of products within the donor vector sequence (CL150/CL116 and CL098/CL134), but none with amplification of the correct knocked-in tagged sequence (i.e. absence of specific bands with CL133/CL132 and CL098/CL138).

C) Representative image of a copGFP-positive clone, showing co-localization of PC1-CTT-copGFP fusion and a mitochondrial marker.

Figure S5. Optimization methods for immunofluorescent detection of mPC1-4119-3HA.

A) Immunofluorescence of NIH3T3 cells expressing mPC1-4119-3HA permeabilized in 0.5% Triton X 100 by HA antibody shows co-localization of HA signal with MitoTracker Deep Red. Treatment with a proteasome inhibitor (MG132) does not affect the distribution pattern.

B) Immunofluorescence of NIH3T3 cells expressing mPC1-4119-EGFP-3HA permeabilized with either 0.1% or 0.5% Triton X 100. An unambiguous mitochondrial pattern of HA antibody staining was revealed only when cells were permeabilized in 0.5% Triton X 100.

Figure S6. Original immunoblots for Figure 6.

A) Immunoblot in Figure 6B, showing the same membrane captured with low (left) and high (right) intensity.

B) Original immunoblot for Figure 6C.

C) Original immunoblot for Figure 6E.

Figure S7. Mitochondrial biomass in transgenic flies expressing PC1-CTT-eGFP is the same as in control flies.

Immunoblotting of several mitochondrial proteins isolated from transgenic flies expressing either mitoGFP or PC1-CTT-eGFP.

SUPPLEMENTARY METHODS.

Lipid extraction and analysis

Sub-confluent cell monolayers in 10-cm dishes were incubated in 10 ml of DMEM/F-12 medium containing 50 μCi of [^{3}H] acetate at 37°C for 24 hour. After washing by PBS with 1 mM EDTA, cells were detached in 1 ml of Accutase (Innovative Cell Technologies, Inc. Cat# AT104-500) at 25°C. Lipids were extracted from the cells as previously described [3]. Phospholipids were separated by thin layer chromatography (TLC) as described [4]. TLC plates were scanned on a RITA Star Thin Layer Analyzer (Raytest). GINA Star TLC was used to compare the sizes and intensities of different lipid spots on the TLC plates among the conditions. By using GINA Star TLC, we obtained integrated density plots for each lane on the TLC plate, whereby each lipid spot corresponded to a peak on the plot. We measured the area of each peak by extrapolating its Gaussian shape to the baseline. The sum of all peaks in a lane corresponded to total lipids in that sample, and each different lipid spot was represented as a percentage of

total lipids in that lane. Differences in lipid profiles among conditions were analyzed with a T test.

Western blot

Immunoblotting was carried out following the standard protocol. Briefly, whole flies were homogenized in RIPA buffer supplemented with protease inhibitors. Total protein concentration was measured by the BCA method (Thermo Fisher Scientific). Equal amount of total protein was subjected to SDS-PAGE. Primary antibodies were used against CoIV (ab16056, Abcam), actin (C4, Millipore), SOD2 (NB100-1992, Novus Biologicals), ATP-synthase 5α (15H4C4, Abcam), and GFP (A-11120, Thermo Fisher Scientific). HRP-conjugated anti-mouse and anti-rabbit secondary antibodies were used (Sigma). SuperSignal West Dura Chemiluminescent Substrate (Thermo Fisher Scientific) was used as a developer. Imaging was recorded by ImageQuant LAS4000 (GE Healthcare) and analyzed by ImageQuant TL software.

REFERENCES

1 Yang, M., Kpalma, K. & Ronsin, J. in *Pattern Recognition Techniques* (ed Peng-Yeng Yin) 43-90 (IN-TECH, 2008).

2 Westrate, L. M., Drocco, J. A., Martin, K. R., Hlavacek, W. S. & MacKeigan, J. P. Mitochondrial morphological features are associated with fission and fusion events. *PLoS One* **9**, e95265, doi:10.1371/journal.pone.0095265 (2014).

3 Bligh, E. G. & Dyer, W. J. A rapid method of total lipid extraction and purification. *Can J Biochem Physiol* **37**, 911-917, doi:10.1139/o59-099 (1959).

4 Vaden, D. L., Gohil, V. M., Gu, Z. & Greenberg, M. L. Separation of yeast phospholipids using one-dimensional thin-layer chromatography. *Anal Biochem* **338**, 162-164, doi:10.1016/j.ab.2004.11.020 (2005).

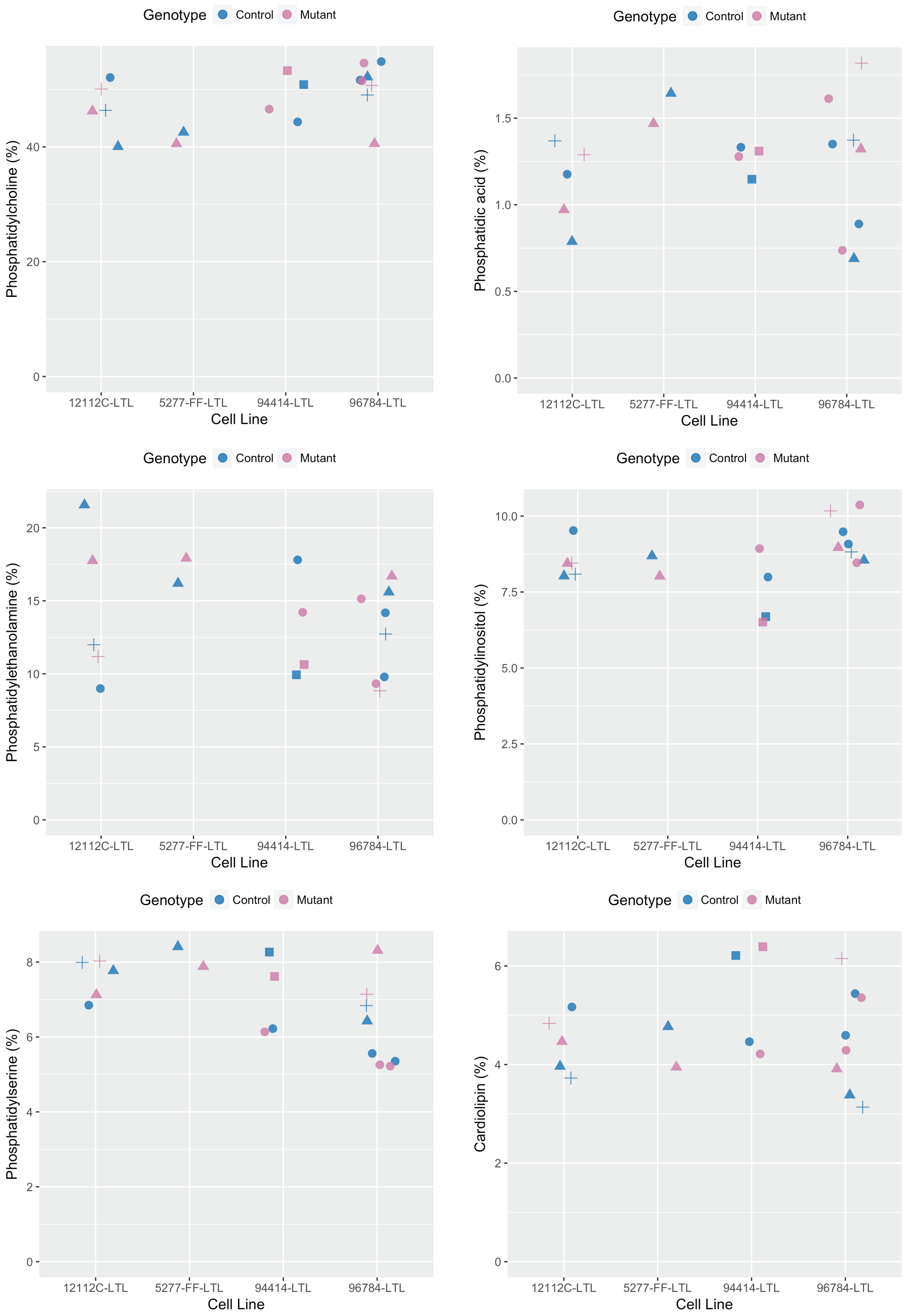
Genotype
Control
Mutant
Phosphatidylcholine (%)
Phosphatidic acid (%)
Phosphatidylethanolamine (%)
Phosphatidylinositol (%)
Phosphatidylserine (%)
Cardiolipin (%)
12112C-LTL
5277-FF-LTL
94414-LTL
96784-LTL
Cell Line

Figure S1

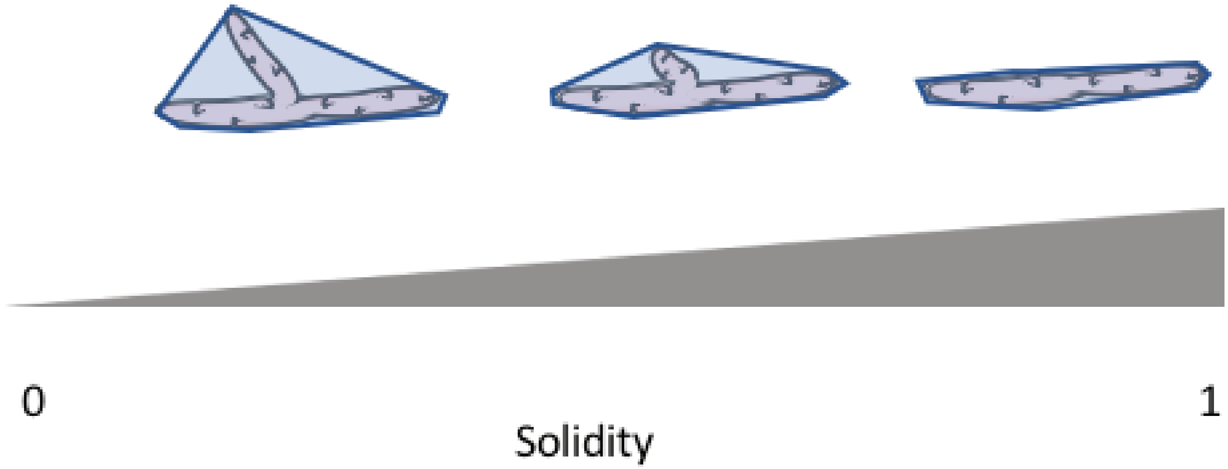

Figure S2

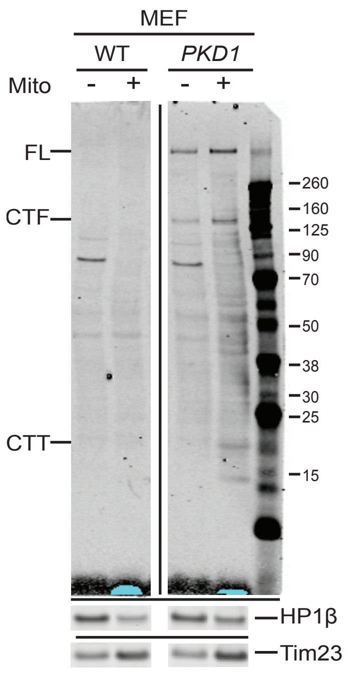
MEF
WT
PKD1
Mito
-
+
-
+
FL
CTF
CTT
260
160
125
90
70
50
38
30
25
15
HP1β
Tim23

Figure S3

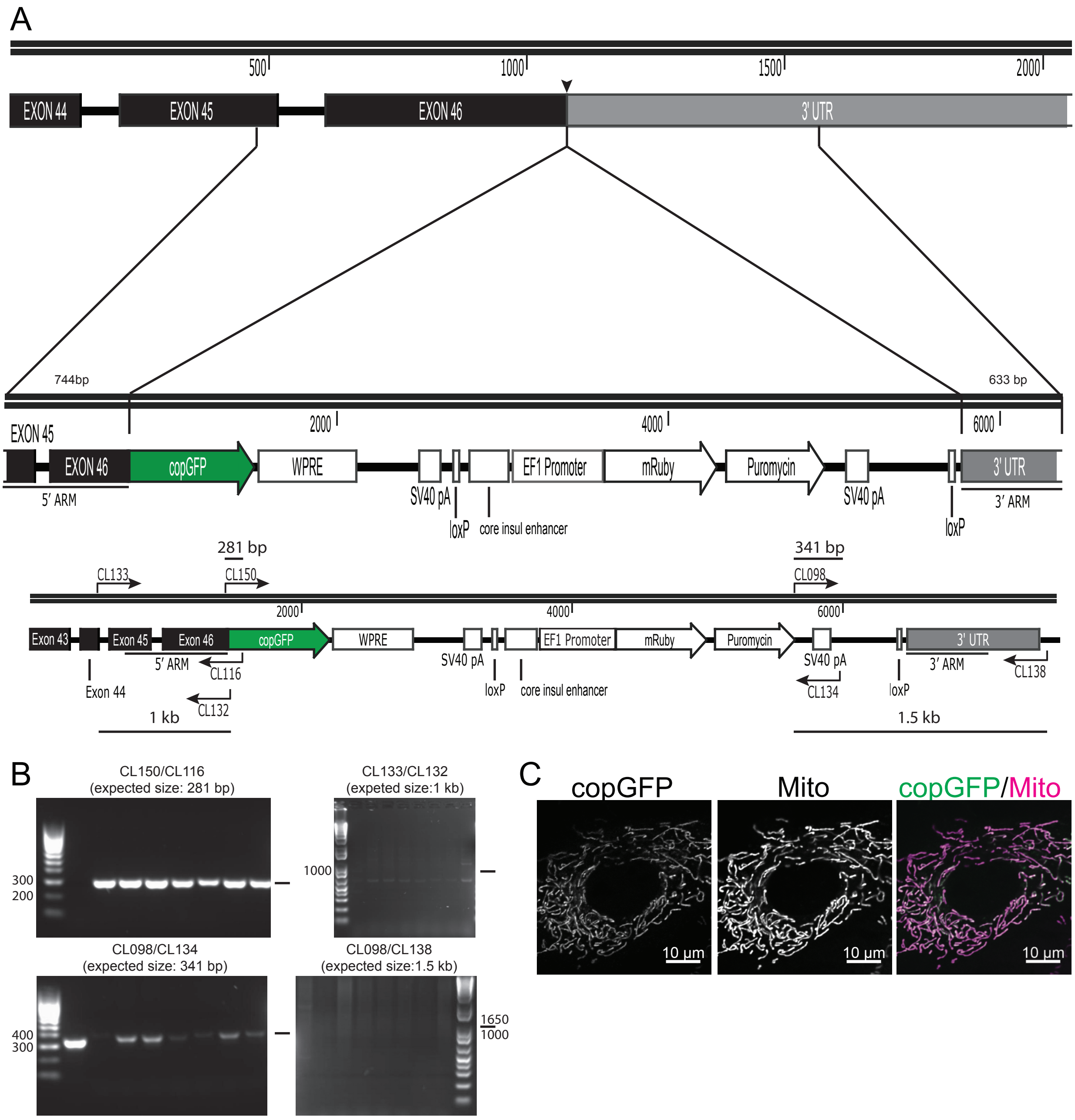
A
500
1000
1500
2000
EXON 44
EXON 45
EXON 46
3' UTR
744bp
633 bp
2000
4000
6000
EXON 45
EXON 46
copGFP
WPRE
EF1 Promoter
mRuby
Puromycin
3' UTR
5' ARM
SV40 pA
loxP
core insul enhancer
SV40 pA
loxP
3' ARM
281 bp
341 bp
CL133
CL150
CL098
Exon 43
Exon 45
Exon 46
Exon 44
CL116
CL132
1 kb
CL134
CL138
1.5 kb
B
CL150/CL116
(expected size: 281 bp)
300
200
CL133/CL132
(expeted size:1 kb)
1000
CL098/CL134
(expected size: 341 bp)
400
300
CL098/CL138
(expected size:1.5 kb)
1650
1000
C
copGFP
Mito
copGFP/Mito
10 µm
10 µm
10 µm

Figure S4

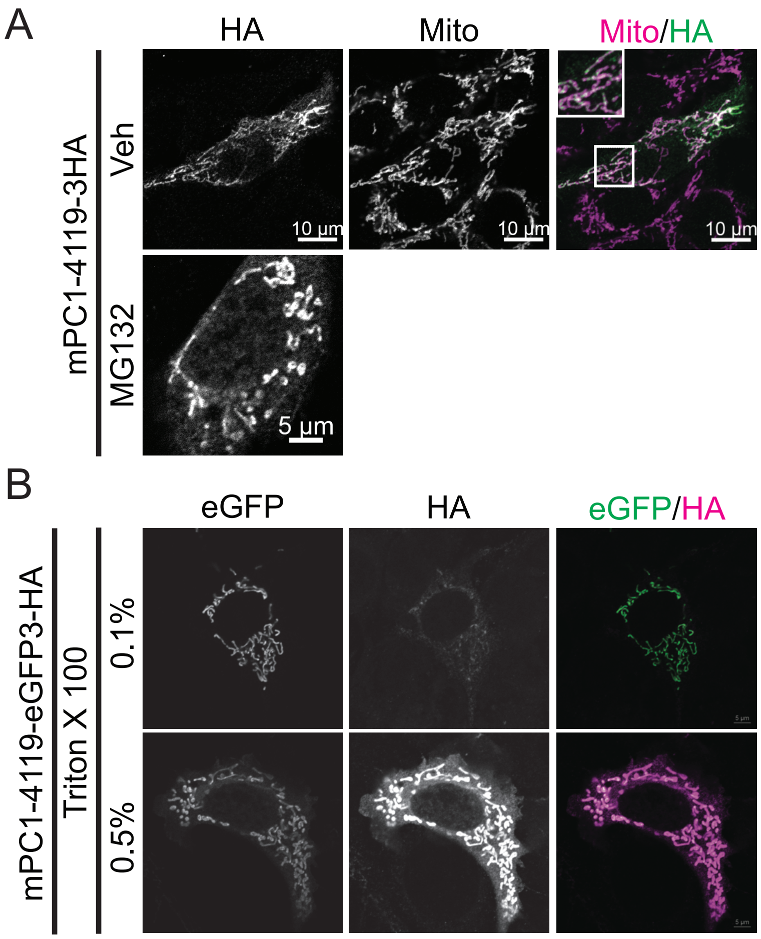
A
HA
Mito
Mito/HA
mPC1-4119-3HA
Veh
MG132
10 µm
10 µm
10 µm
5 µm
B
eGFP
HA
eGFP/HA
mPC1-4119-eGFP3-HA
Triton X 100
0.1%
0.5%

Figure S5

A

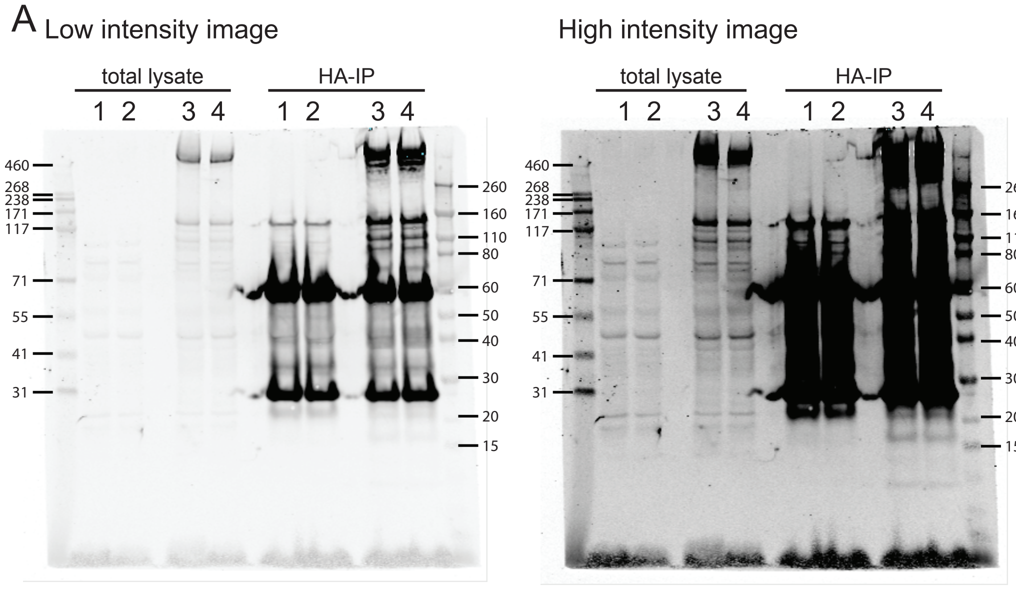

Lane 1, MDCK pcDNA5, base line
Lane 2, MDCK pcDNA5, Hypoxia 2hour
Lane 3, MDCK GFP-PC1-HA,baseline
Lane 4, MDCK GFP-PC1-HA, Hypoxia 2hour

B

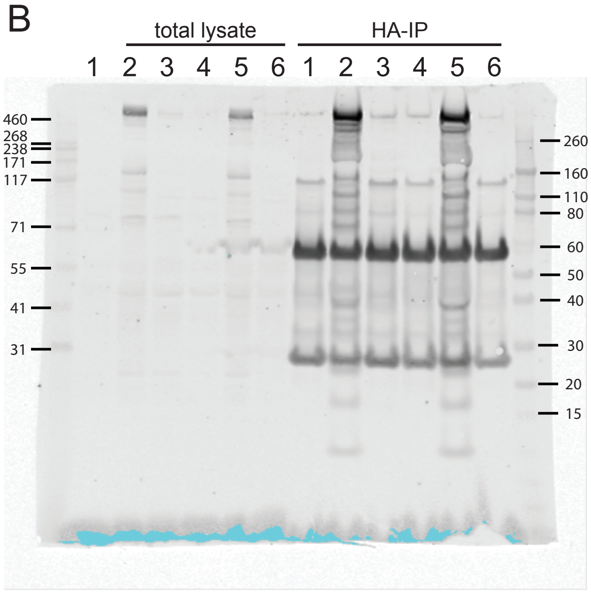

Lane 1, MDCK pcDNA5, baseline
Lane 2, MDCK GFP-PC1-HA, baseline
Lane 3, MDCK GFP-PC1-HA, complete starvation for 8 hours
Lane 4, MDCK GFP-PC1-HA, complete starvation for 8 hours (dead/floating cells)
Lane 5, MDCK GFP-PC1-HA, serum starvation for 48 hours
Lane 6, MDCK GFP-PC1-HA, serum starvation for 48 hours (dead/floating cell)

C

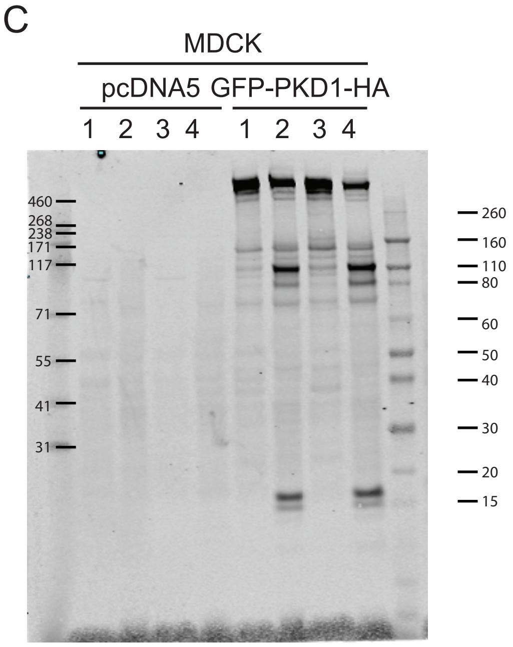

Lane 1, total lysate
Lane 2, mitochondrial fraction
Lane 3, total lysate, 50uM DAPT
Lane 4, mitochondrial fraction, 50uM DAPT

Figure S6

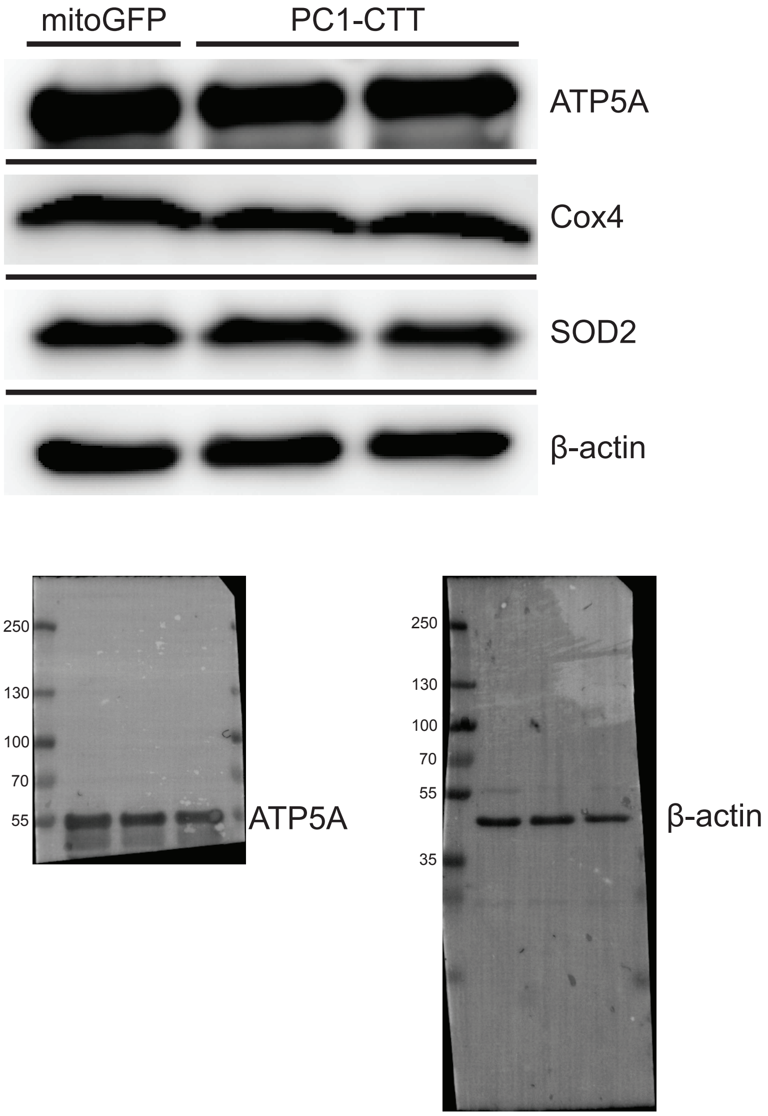
mitoGFP
PC1-CTT
ATP5A
Cox4
SOD2
β-actin
250
130
100
70
55
ATP5A
250
130
100
70
55
35
β-actin

Figure S7
